# Non‐Covalent Integration of a [FeFe]‐Hydrogenase Mimic to Multiwalled Carbon Nanotubes for Electrocatalytic Hydrogen Evolution

**DOI:** 10.1002/chem.202202260

**Published:** 2022-10-19

**Authors:** Afridi Zamader, Bertrand Reuillard, Jacques Pécaut, Laurent Billon, Antoine Bousquet, Gustav Berggren, Vincent Artero

**Affiliations:** ^1^ Univ. Grenoble Alpes CNRS CEA IRIG Laboratoire de Chimie et Biologie des Métaux 17 rue des Martyrs F-38054 Grenoble, Cedex France; ^2^ Univ. Grenoble Alpes CEA CNRS IRIG-SyMMES UMR 5819 38000 Grenoble France; ^3^ Universite Pau et des Pays de l'Adour E2S UPPA CNRS IPREM 64000 Pau France; ^4^ Bio-inspired Materials Group: Functionalities & Self-Assembly Universite de Pau et Pays de l'Adour E2S UPPA 64053 Pau France; ^5^ Molecular Biomimetics Department of Chemistry – Ångström Laboratory Uppsala University Box 523 SE-75120 Uppsala Sweden

**Keywords:** carbon nanotubes, hydrogenase mimic, hydrogen evolution reaction, molecular electrocatalysis, pyrene.

## Abstract

Surface integration of molecular catalysts inspired from the active sites of hydrogenase enzymes represents a promising route towards developing noble metal‐free and sustainable technologies for H_2_ production. Efficient and stable catalyst anchoring is a key aspect to enable this approach. Herein, we report the preparation and electrochemical characterization of an original diironhexacarbonyl complex including two pyrene groups per catalytic unit in order to allow for its smooth integration, through π‐interactions, onto multiwalled carbon nanotube‐based electrodes. In this configuration, the grafted catalyst could reach turnover numbers for H_2_ production (TON_H2_) of up to 4±2×10^3^ within 20 h of bulk electrolysis, operating at neutral pH. *Post operando* analysis of catalyst functionalized electrodes revealed the degradation of the catalytic unit occurred via loss of the iron carbonyl units, while the anchoring groups and most part of the ligand remained attached onto multiwalled carbon nanotubes.

## Graphical abstract

**An original [FeFe]‐hydrogenase mimic** integrating two pyrene anchors was successfully synthesized and grafted at the surface of multiwalled carbon nanotube based electrodes. The hybrid molecular cathode could reach high turnover numbers for H_2_ production in fully aqueous media and sustain activity for 20 h.

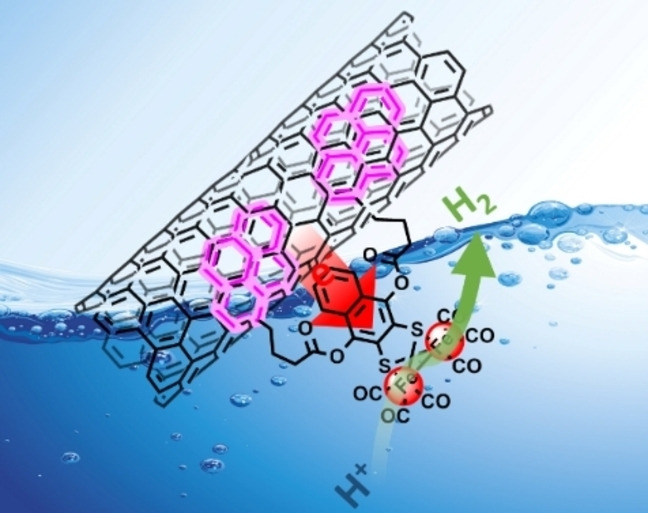

## Introduction

(Photo)‐electro catalytic water splitting provides a method to store intermittent solar energy under the form of chemical bonds (H−H). Releasing only H_2_O and heat as side products, when oxidized at the anode of a fuel cell to generate electricity, solar hydrogen is considered as a promising “green” energy buffer.[^1^, ^2^, ^3^] However, to become broadly available, direct water electrolysis devices should ideally rely on inexpensive, durable and efficient catalysts.[^4^, ^5^, ^6^] As of today, platinum group metals (PGMs) remain the benchmark catalysts for electrolyzers design due to their high catalytic activity at low overpotential and considerable stability, but the scalability of PGMs remains a major constrain.[^7^, ^8^, ^9^, ^10^, ^11^]

Besides PGMs, hydrogenase (H_2_ase) enzymes have attracted substantial attention in this context as they are capable of oxidizing and producing H_2_ at outstanding catalytic rates (6000–9000 s^−1^).^[12]^ Remarkably, these activities are achieved at low overpotential and neutral pH, using only earth abundant metals, i. e. Ni and/or Fe.[^12^, ^13^, ^14^, ^15^, ^16^] Among hydrogenases, the [FeFe]‐hydrogenases ([FeFe]‐H_2_ases) stand out as the most active for hydrogen evolution.[^12^, ^16^] Despite the large diversity among [FeFe]‐H_2_ases, they all feature a common active site called the “H‐cluster”.^[16]^ X‐ray crystallography in combination with spectroscopy and model chemistry studies have revealed that the H‐cluster features a unique low‐valent diiron subsite ([FeFe]), with a bridging azadithiolate ligand (ADT^2−^, ^−^SCH_2_NCH_2_S^−^). The Fe ions of the [FeFe] subsite are further coordinated by one terminal CO and one terminal CN^−^ ligand each, and share an additional bridging CO ligand.[^17^, ^18^, ^19^, ^20^, ^21^] In addition, the [FeFe] subsite is covalently bound to a canonical [4Fe4S] cluster through a thiolate ligand from a bridging cysteine residue. The protein matrix around the H‐cluster provides multiple functionalities essential for the overall catalytic performance (e. g. controlling substrate access, providing site isolation and water solubility, stabilising reactive entatic states, etc.).[^22^, ^23^] However, their sensitivity to many operational conditions and large molecular footprint (diameter ∼5–10 nm) limit their integration to functional devices.[^24^, ^25^] Nevertheless, hydrogenases have served as valuable blueprints for the design of noble metal‐free bioinspired catalysts for hydrogen production and oxidation.[^26^, ^27^, ^28^, ^29^, ^30^, ^31^] Among such bioinspired catalysts, hexacarbonyl‐ dithiolato‐diiron systems of the general structure {(μ‐S_2_)Fe_2_(CO)_6_} remain among the most studied for photo‐ and electrochemical hydrogen production.^[32]^ However, due to their poor water solubility there is limited information available on their activity in aqueous media, an essential requirement for sustainable H_2_ production.[^33^, ^34^] Instead, these iron carbonyl complexes have primarily been studied under homogenous electrochemical conditions in organic media.^[35]^ This resulted in performances limited by low diffusion rates and degradation processes during catalysis through self‐association of reactive intermediates.[^36^, ^37^] Moreover, in order to shift from basic science to applications, molecular electrocatalysts need to be heterogenized at electrode surfaces.[^38^, ^39^, ^40^, ^41^] This development towards applications not only provides a path to circumvent the aforementioned limitations but also enables additional control over multiple important parameters influencing catalysis,^[38]^ such as catalyst concentration at materials‐electrolyte interface,^[42]^ electrode‐catalyst electron transfer rate,^[43]^ catalyst activity,[^43^, ^44^] selectivity[^45^, ^46^] and allows to operate in aqueous condition even for water insoluble catalyst.^[34]^

Over the past two decades, there have been multiple examples of {(μ‐S_2_)Fe_2_(CO)_6_} derivatives immobilized at electrode surfaces but only a few of them showed sustained activity in aqueous conditions.[^47^, ^48^, ^49^, ^50^] Recently, Dey et al. reported electrocatalytic activity of water insoluble diiron complexes under fully aqueous conditions, following physisorption on edge plane graphite electrodes (EPGs), with some of these complexes showing good oxygen tolerance or even bidirectionality for H_2_ production and oxidation.[^34^, ^51^, ^52^] One of these molecular systems reached a high turnover frequency (TOF_max_) of 6400 s^−1^ at −0.5 V vs. NHE in 0.5 N H_2_SO_4_.^[34]^ The relatively low overpotential requirement was attributed to the stabilization of reduced catalytic intermediates in aqueous media due to solvation. The same group also reported the covalent attachment of an analogous {(μ‐S_2_)Fe_2_(CO)_6_} complex on EPGs and graphene oxide (GO) electrodes.^[53]^ In parallel, the immobilization of various related [FeFe]‐H_2_ase mimics on multi‐walled carbon nanotube (MWNT) functionalized electrodes has been shown to provide remarkable stability and activity under acidic aqueous condition.[^54^, ^55^] Among the previously reported grafting strategies, immobilization relying on nonspecific physisorption often results in poor electron transfer rates combined with limited stability of the anchoring over long term electrolysis,^[56]^ while covalent coupling typically leads to lower surface loadings,[^47^, ^53^] inhomogeneous catalyst distribution and possible degradation of the MWNT support.^[57]^ In recent years, non‐covalent integration of metal complexes modified with pyrene units onto *π*‐conjugated supports, using *π‐π* interactions, has been shown to improve catalyst loading and the electronic communication between the complex and the electrode, while providing stable grafting in aqueous catalytic conditions.[^46^, ^58^, ^59^, ^60^, ^61^, ^62^, ^63^, ^64^, ^65^] To date, this mild surface functionalization strategy has not yet been reported for the anchoring of {(μ‐S_2_)Fe_2_(CO)_6_} derivatives.

Herein, we explore the feasibility of the latter approach. We report the synthesis and electrochemical characterization of a new pyrene‐bearing diiron hexacarbonyl complex, as well as its electrocatalytic performance following non‐covalent grafting onto MWNT‐based electrodes.

## Results and Discussion

**Scheme 1:**
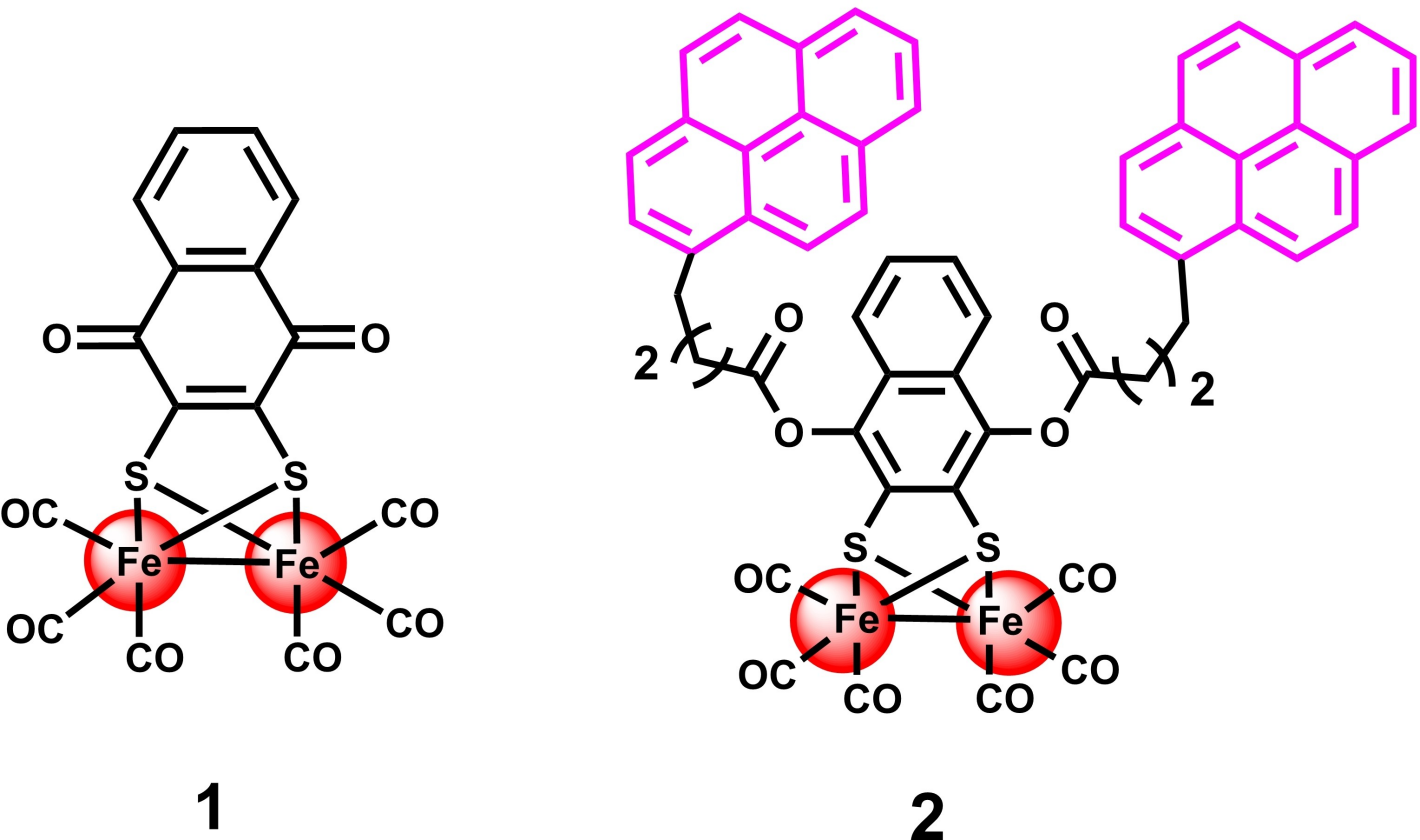
Schematic representation of complex **1** (synthetic precursor) and complex **2** (pyrene functionalized)

**Synthesis and characterization of complexes 1 and 2**. The bioinspired [FeFe]‐hydrogenase mimic **1** ([(μ‐1,4‐naphthoquinone‐2,3‐dithiolato)Fe_2_S_2_(CO)_6_]_,_ (Scheme 1, left) was first synthesized using a previously reported protocol.^[66]^ Subsequently, complex **2** (Scheme 1, right) was synthesized via a nucleophilic substitution reaction between chemically reduced complex **1** and commercially available 1‐pyrenebutyric acid *N*‐hydroxysuccinimide ester (Scheme S1). High resolution mass spectroscopy (HRMS) further confirmed the exact mass of complex **2** (Figure S1).

**Figure 1:**
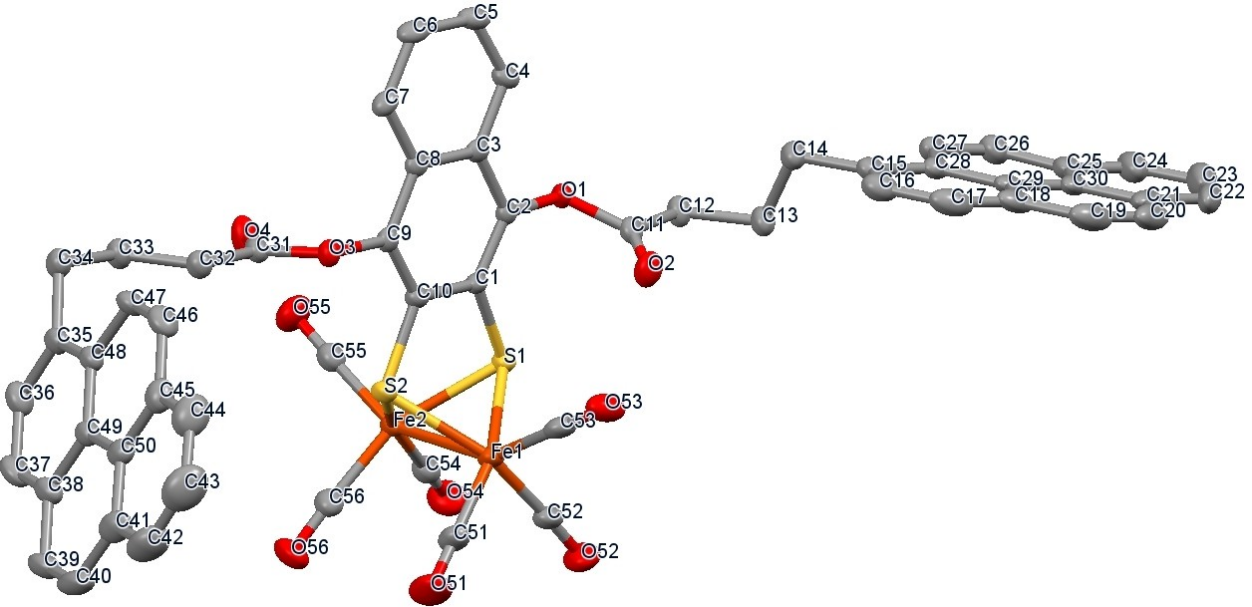
X‐ray crystal structure of complex **2**. Color coding: Fe orange; S yellow; C grey; and O red. Hydrogen atoms are omitted for clarity. Thermal ellipsoids set at 50 % probability level. Selected bond distance (Å) and angles (^0^): Fe1−Fe2, 2.4802(5); Fe1−S1, 2.2598(8); Fe1−S2, 2.2776(8); Fe1−C51, 1.791(3); Fe1−C52, 1.789(3); Fe1−C53, 1.805(3); Fe2−S1, 2.2678(7); Fe2−S2, 2.2743(7); Fe2−C54, 1.793(3); Fe2−C55, 1.806(3); Fe1−C56, 1.796(3); S1−Fe1−S2, 81.24(3); S1−Fe2−S2, 81.13(3). CCDC 2183292 contains supplementary crystallographic data. The assymetric unit also contains two co‐crysatllized solvent molecules (CH_2_Cl_2_) which were removed from the figure for better clearity.

The X‐ray single crystal structure of complex **2** revealed a triclinic structure in which the two pyrene units are orthogonal to each other. The Fe−Fe, Fe−S, Fe−CO bond distances were similar to that of related {(μ‐S_2_)Fe_2_(CO)_6_} complexes (see crystallographic data, Tables S6—S10).^[67]^ The crystal structure also showed intermolecular interactions between the organometallic cores of the two complexes, generating a dimeric structure (Figures 1 & S2).

**Figure 2:**
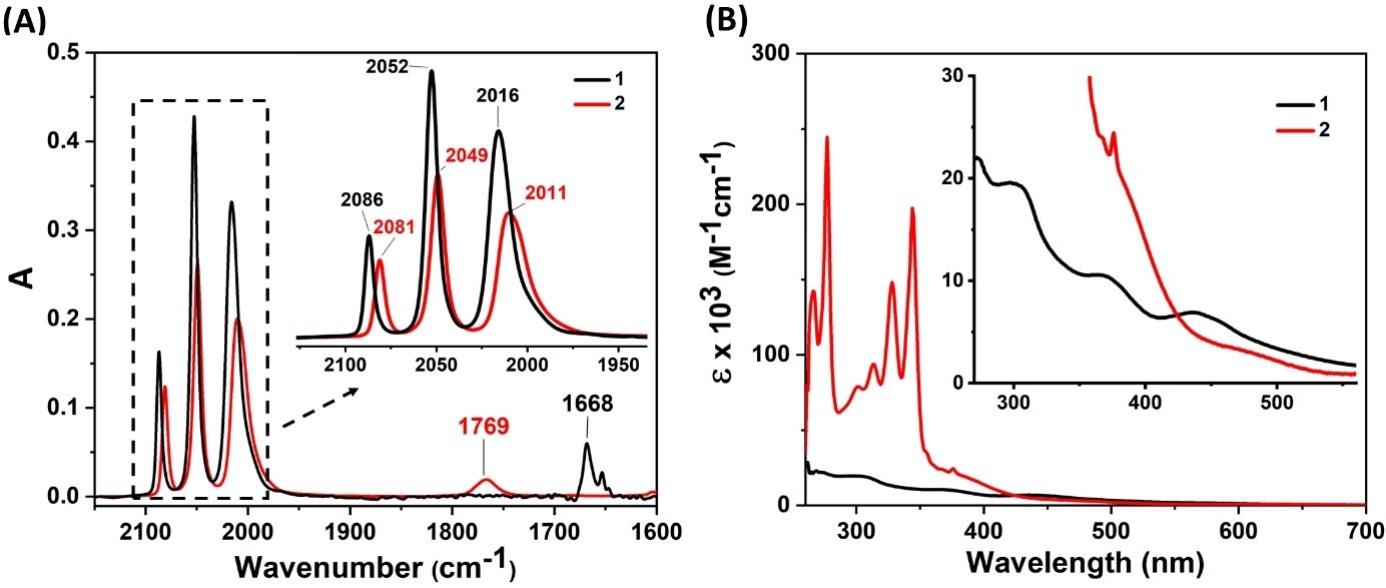
Infrared and UV‐Vis spectra of **1** and **2**. (A) Infrared spectra of complex **1** (black trace) and **2** (red trace), concentrations 3 mM in CHCl_3_; (B) UV‐Vis spectra of **1** (black trace) and **2** (red trace), concentrations 3.2 μM in DMF.

Complexes **1** and **2** were analyzed and compared in solution through a combination of NMR, UV‐Vis, FTIR and fluorescence spectroscopy. A comparison of the ^1^H NMR peak position of the bridgehead naphthalene group protons between **2** and **1** revealed an upfield shift of the protons in complex **2** (Figures S3 and S4); in line with an increase of the electron density of the aromatic group in complex **2** following the conversion of the quinone carbonyls into ester functional groups. Infrared spectroscopy allowed observing the characteristic CO stretching peaks of the complexes. Three distinct peaks were observed for **1**, at 2086, 2052 and 2016 cm^−1^. The overall shape of the spectrum remained unaltered upon formation of **2**, but the peaks shifted to 2081, 2049 and 2011 cm^−1^. This bathochromic shift of the carbonyl bands in complex **2** also reveals a slightly higher electron density at the diiron center (Figure 2A), as expected from a more electron rich bridging ligand. In addition, a new peak observed at 1769 cm^−1^ was attributed to the carbonyl bond of the ester group for **2**, appearing with concomitant loss of a peak at 1668 cm^−1^ for **1** attributed to the carbonyl bonds of the quinone (Figure 2A). UV‐Vis spectra of complex **2** also displayed characteristic pyrene absorptions with sharp peaks between 255–350 nm observed for complex **2** unlike complex **1** (Figure 2B, red trace). Two additional peaks observed around 371–435 and 435–540 nm for both **1** and **2** were attributed to the metal to ligand charge transfer (MLCT) involving Fe to CO and Fe to S ligands, respectively.^[68]^ Fluorescence spectroscopy revealed the absence of peak around 500 nm, that would otherwise suggest inter/intra‐molecular interactions between pyrenes to form excimers (Figure S6).^[69]^ Therefore, the distance between the pyrene rings in solution exceeds 5 Å, the minimum distance required to form excimer between pyrenes.^[69]^

**Figure 3:**
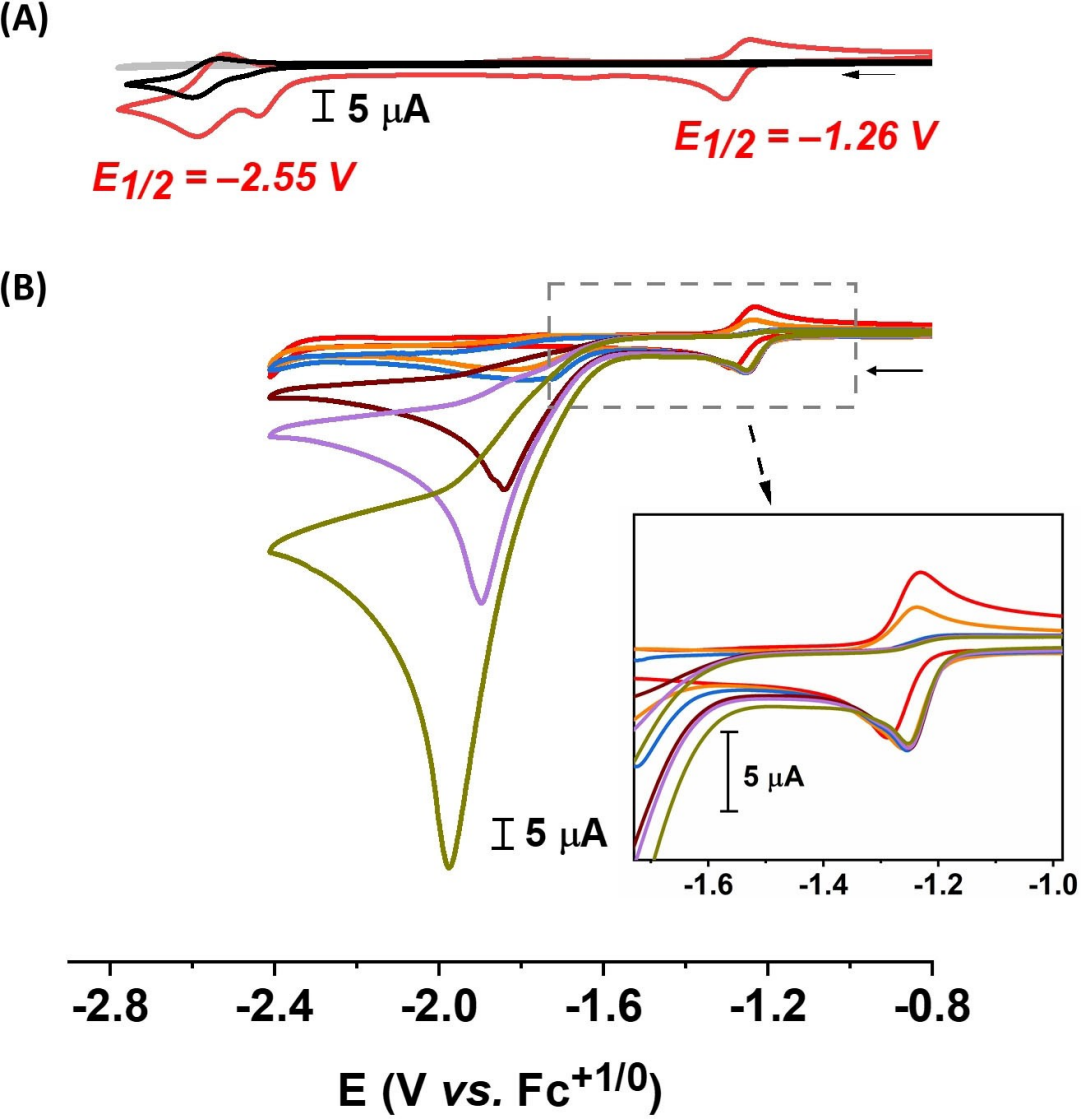
CV traces of **2** (1 mM) under homogenous condition. (A) Comparison between blank (grey trace), **2** (red trace) and 1‐pyrenebutyric acid (black trace); (B) **2** in the presence of different concentrations of trifluoroacetic acid (red=0 mM, orange=1 mM, violet=2 mM, brown=5 mM, purple=10 mM, dark yellow=20 mM). Inset showcases the non‐turnover signals as well as onset potentials at different acidic condition. All CV traces were recorded in DMF, with 0.1 M TBAPF_6_, at room temperature, ν=100 mV s^−1^.

**Electrochemical characterization**. Due to its relatively poor solubility in acetonitrile, the electrochemical properties of complex **2** were characterized in DMF. Under these conditions, complex **2** displayed a quasi‐reversible redox event at E_1/2_=−1.26 V vs Fe^+/0^ (with a peak separation, E_p,c_−E_p,a_=ΔE_p_=60 mV) (Figure 3A, red trace). We tentatively attribute this process to a two‐electron reduction of the complex (Fe^I^Fe^I^/Fe^0^Fe^0^), in line with closely related systems featuring benzene‐dithiolate (bdt^2−^) bridging ligands, for which a single step two‐electron reduction has been characterized.[^70^, ^71^, ^72^, ^73^] The peak currents of the redox process were found to vary linearly with the square root of the scan rate (from 10 to 1000 mV s^−1^), indicating a diffusion controlled redox event (Figure S8).^[74]^ At more reducing potentials, an irreversible reduction at E_p,c_=−2.44 V vs Fe^+/0^ was observed, followed by a (quasi‐) reversible process at E_1/2_=−2.55 V vs Fe^+/0^ (ΔE_p_=78 mV). The former process cannot be assigned with certainty and can be attributed to either the Fe−CO core or the bridging ligand, while the latter is assigned to two single electron processes from the two pyrene groups of complex **2**, matching that observed for 1‐pyrenebutyric acid and as previously reported^[58]^ (Figure 3A, black trace).

Addition of trifluoracetic acid (TFA) to 1 mM solution of complex **2** leads to a 40 mV anodic shift with a moderate loss of reversibility of the first reduction event (Figure 3B).[^66^, ^73^] The decrease in reversibility already observed at stoichiometric amounts of TFA (Figure 3B inset) indicates that the reduced intermediate readily reacts with protons. The same behavior has been reported by others for structurally related [Fe_2_(μ‐bdt)(CO)_6_] complexes.[^66^, ^73^, ^75^] The disapearance of the (re‐)oxidation wave and anodic shift of the reduction peak is thus attributed to the protonation of the doubly reduce diiron complex, likely forming a μ‐hydride species which then requires an extra electron at lower potential (see below) to generate the H_2_ forming species.^[73]^ Moreover, the addition of increasing amounts of TFA gave rise to a new reduction process with an onset potential of about −1.6 V vs Fe^+/0^ (Figure 3B). Increasing the acid concentration resulted in a gradual increase in current to reach up to 90 μA at −1.98 V vs Fe^+/0^ in the presence of 20 equivalents of TFA (20 mM, Figure 3B, dark yellow trace). Together, these results suggest that **2** is capable of catalytic proton reduction, as reported for the related [Fe_2_(μ‐bdt)CO_6_] complex.[^66^, ^73^, ^75^]

**Figure 4:**
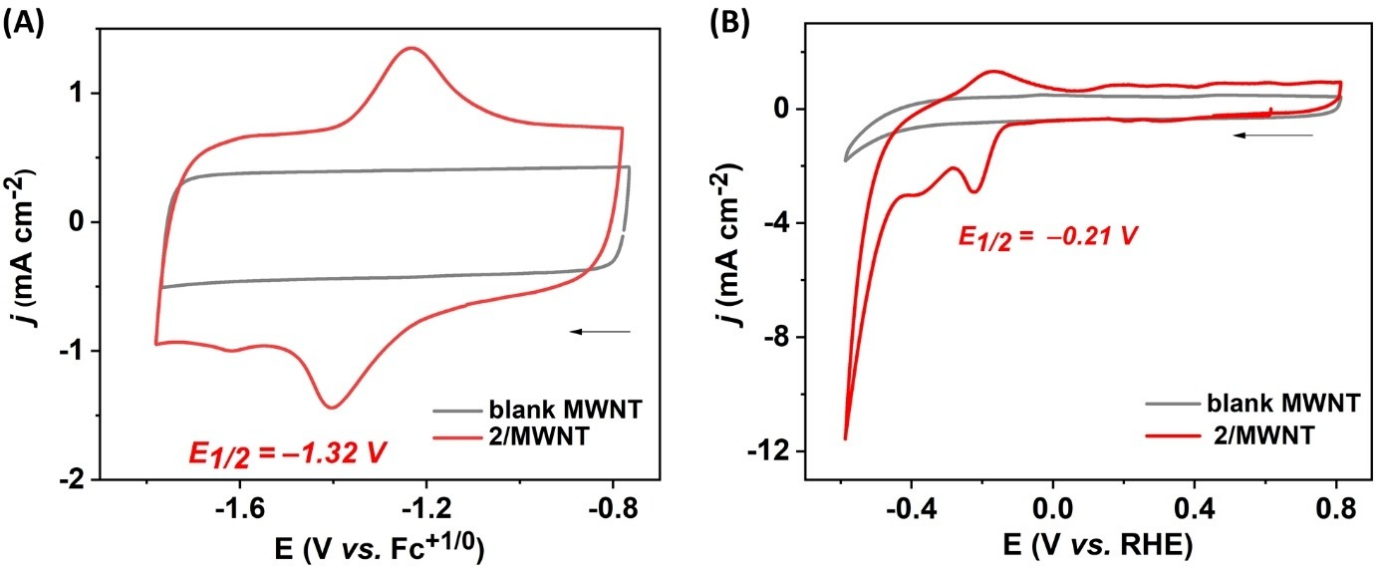
CV traces of the immobilized catalyst (**2**) in organic and aqueous media. (A) CV traces of complex **2**/MWNT (red trace) and the blank MWNT electrode (grey trace), recorded in acetonitrile with 0.1 M TBAPF_6_; and (B) CV traces of **2**/MWNT (red trace) and the blank MWNT electrode (grey trace) recorded in 0.2 M sodium phosphate buffer of pH 7. All CV traces collected at room temperature, ν=100 mV s^−1^.

Taking advantage of the pyrene units, complex **2** was subsequently grafted on multiwall carbon nanotubes (MWNT) by soaking the modified electrode in a solution of complex **2** in DMF for 10 min before rinsing it to remove any unbound species (see Figure S9 for a schematic description). CVs of the modified electrode (**2**/MWNT) recorded in acetonitrile revealed a (quasi‐) reversible redox process at E_1/2_=−1.32 V vs Fe^+/0^ (Figure 4A). The peak currents of **2**/MWNT showed linear correlations with scan rate (10–100 mV s^−1^) as expected from immobilized species on the electrode surface (Figure S10). This redox event was attributed to the same two‐electron redox process albeit slightly shifted towards more reducing potentials as compared to what was observed under homogenous condition (Table S1), which is likely due to the change of solvent (acetonitrile vs. DMF) between the homogeneous and heterogeneous experiment combined with the change of chemical environment of the complex upon grafting at the electrode surface. By comparison, physisorbed complex **1** onto MWNT displayed an analogous (quasi‐) reversible 2e^−^ redox process at E_1/2_=−1.18 V vs Fe^+/0^. The positive shift (∼140 mV) of the redox event relative to **2**/MWNT is attributed to the presence of relatively strong electron withdrawing quinone functionalities in the bridgehead for complex **1** as explained earlier (Figure S11).

Under fully aqueous conditions (0.2 M sodium phosphate buffer at pH 7), CVs of **2**/MWNT showed two distinct redox processes. A quasi‐reversible (ΔE_p_=48 mV) redox process at E_1/2_=−0.21 V vs. RHE (reversible hydrogen electrode) and an irreversible reduction at E_p,c_=−0.36 V vs. RHE attributed to the Fe^I^Fe^I^/Fe^I^Fe^0^ and Fe^I^Fe^0^/Fe^0^Fe^0^ redox couples respectively (Figure 4B). Considering the small separation between the reduction events, the initially reduced species could arguably be stabilized via a structural change, most likely protonation. A catalytic reduction wave, corresponding to H_2_ evolution (see below) could also be observed at an onset potential of about −0.44 V vs. RHE, which will be further discussed below. CV traces of **1**/MWNT, prepared analogously to **2**/MWNT, were recorded to probe the effect of the pyrene units on performance. As expected from the proposed anchoring model, both the stoichiometric and catalytic currents for **1**/MWNT were smaller as compared to **2**/MWNT (Figure S12), indicating that **1** can bind to MWNT through nonspecific interactions but affording a relatively low loading. The loading of **2** on MWNT was estimated from the CV traces and spectroscopy. Integration of the oxidation wave of the system observed at E_1/2_=−1.32 V vs. Fc^+/0^ in acetonitrile (Figure 4A), indicated 7.6±0.9 nmol cm^−2^ of electroactive catalyst at the electrode, assuming a Fe^I^Fe^I^/Fe^0^Fe^0^ redox event. Independently, a catalyst loading of 9.5±0.1 nmol cm^−2^ was determined for complex **2**/MWNT using UV‐Vis spectroscopy following catalysts desorption in DMF, calculated based on the peak at 345 nm (*ϵ*_345nm_=1.95×10^5^ M^−1^ cm^−1^) (Figure 2B, Figure 6B & Table S2). These observations suggest that about 80 % of the total amount of grafted complex **2**, is redox active. Similarly, integration of the redox wave at E_1/2_=−1.18 V vs. Fc^+/0^ for complex **1**/MWNT allowed to quantify 4.5±0.6 nmol cm^−2^ of active sites on the electrode, which further confirms the relatively poorer active site loading using physisorption (Figure S11). The pyrene‐based functionalization strategy thus enables efficient catalyst grafting with ∼2 times higher loading compared to physisorption and up to 30 and 240 times higher loading of electro active sites than what has been reported for analogous [FeFe] complexes covalently attached (0.24 nmol cm^−2^)^[53]^ and physisorbed (0.032 nmol cm^−2^)^[52]^ at EPG electrodes, respectively (Table S2).

**Electrocatalytic production of H_2_ with 2/MWNT**. Scanning towards more negative potentials, a catalytic wave at an onset potential of approximately −0.44 V vs. RHE was observed with the **2**/MWNT modified electrode, reaching current of −11.9±0.9 mA cm^−2^ at −0.6 V vs. RHE (Figure 4B). To assess the stability of the catalytic response as function of the applied potential, repeated CV scans were recorded with the **2**/MWNT modified electrodes within different potential ranges and the change in current amplitude of the non‐turnover signal at −0.21 V was used as a proxy for catalyst stability. Marginal decrease in peak current was observed when scanning exclusively between +0.8 V and −0.03 V vs. RHE (Figure S13A). Expanding the potential window to include the first quasi‐reversible reduction (potential window of +0.8 V to −0.33 V), resulted in a 37–40 % loss of peak intensity (both anodic and cathodic) during the first five cycles (Figures S13B, S14B). Further expanding the potential range (+0.8 to −0.6 V), resulted in even more pronounced decrease of both redox and catalytic responses (Figure S14A, B). The losses mainly occurred during the first five scans before the current plateaued at about 20 % of its initial value (Figure S14B). Thus, degradation of the active sites due to reduction or “reductive inactivation” becomes more pronounced as the potential is scanned towards more negative potentials at which the complex is reduced. The observation that a plateau was reached after the initial reductive inactivation process implies a certain level of inhomogeneity in the binding of **2** to the MWNT, with only a limited fraction displaying sustained activity (Figure S14B).

**Figure 5:**
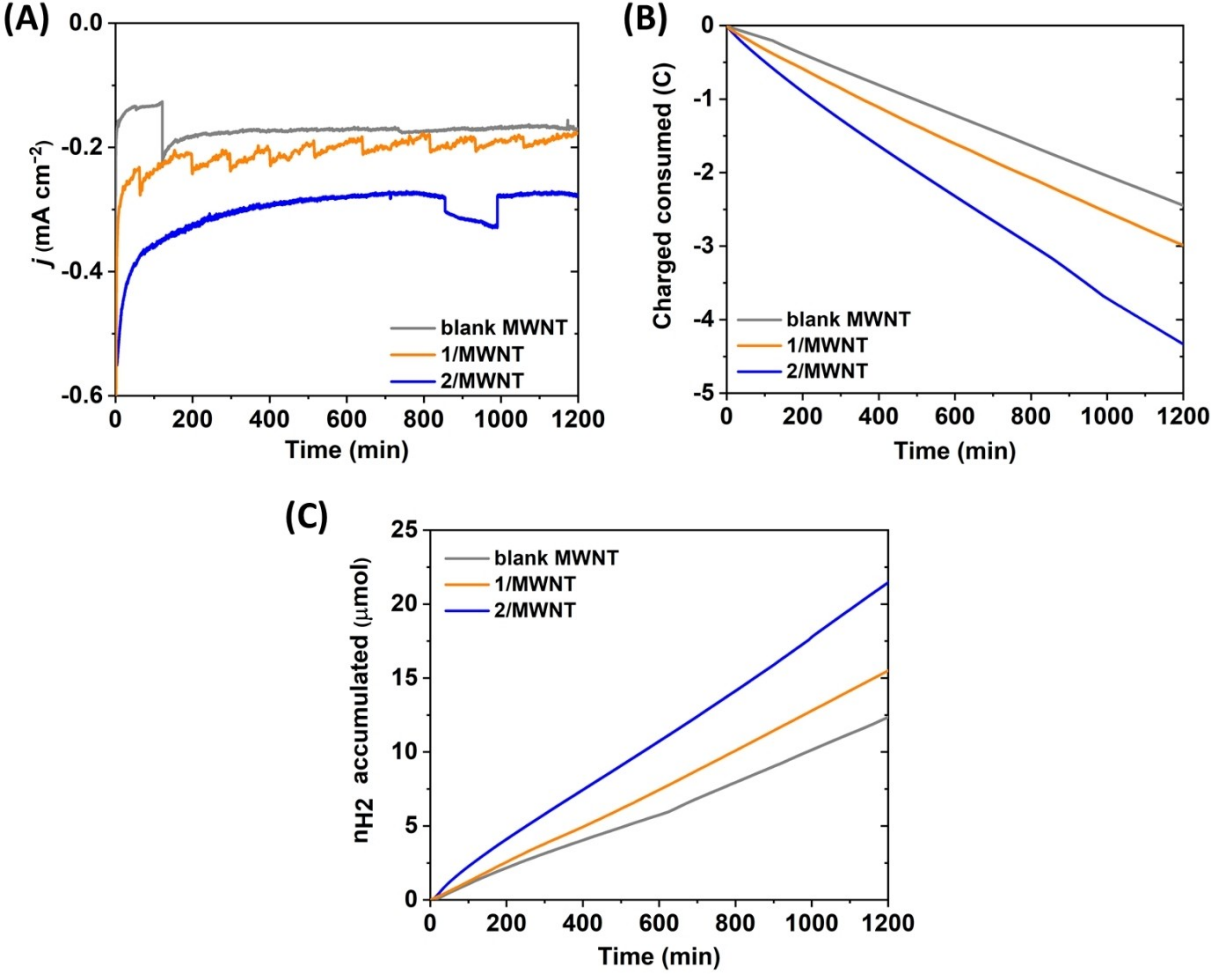
Sustained H_2_ production monitored over 20 h by chronoamperometry, head space gas analysis and consumption of charge of **1**/MWNT (orange trace) **2**/MWNT functionalized electrodes (blue trace) and bare MWNT (grey trace). (A) current density; (B) evolution of the charge; and (C) corresponding production of H_2_, over time during CA performed. Electrodes poised at −0.49 V vs. RHE in 0.2 M sodium phosphate buffer of pH 7 under Argon at room temperature. The fluctuation of current in CA was attributable to bubble formation at the working electrode during electrolysis. See also Figure S15 for replicates for **2**/MWNT.

The CV data were further complemented by chronoamperometry (CA) experiments on the **2**/MWNT electrode to gain further insight on its stability as well as to determine the Faradaic efficiency for H_2_ production (FE_H2_). CA were performed in the same neutral aqueous conditions but using a two‐compartment cell. The electrode was poised at −0.49 V vs. RHE to limit the reductive inactivation while still ensuring relatively large currents. The H_2_ content of the headspace was continuously assessed using gas chromatography (Figure 5 and S15).

For **2**/MWNT, an initial current density of −0.71±0.2 mA cm^−2^ was observed, determined at t=2 min to avoid capacitive current contributions from the MWNT. Indeed, at that point about 27.5 mC of charge was consumed and ∼1 nmol of H_2_ was detected which corresponds to negligible faradic efficiency (FE_H2_) of ≈0.7 %. A further 57 % loss in current was observed during the first three hours, to reach −0.3±0.1 mA cm^−2^ (Figure 5A). The current then remained reasonably stable until the end of the electrolysis (−0.25±0.03 mA cm^−2^). Thus, about 40 % of the initial current was retained over the course of the experiment and remained higher than bare MWNT control electrode (−0.17 mA cm^−2^) during the entire electrolysis. In the case of **1**/MWNT, CA under equivalent conditions showed only moderate activity. An initial current density of −0.42±0.9 mA cm^−2^ (at t=2 min) was obtained which decreased to −0.22±0.02 mA cm^−2^, i. e. a 53 % current loss and very close to that of blank MWNT (−0.175 mA cm^−2^), within three hours. Further extension of the CA lead to a current drop to reach around −0.18±0.01 mA cm^−2^ after 20 h (Figure 5A, Table S3). After 20 h, 15±1 and 19±3 μmol of H_2_ were produced from **1**/MWNT and **2**/MWNT with a FE_H2_ of ≈98–99 %, respectively. This corresponds to 2.2±0.6 and 7±3 μmol of H_2_ and to a TON_H2_ of 2±1×10^3^ and 4±2×10^3^ based on total catalyst loading of **1** and **2**, respectively, after subtraction of the H_2_ produced by blank MWNT (Figure 5, Table S3). We note that the latter values should be considered conservative as direct H_2_ evolution at the MWNT surface may be lower when these are modified with **1** or **2** and that the higher value for the surface concentration of **2** (from UV‐Vis) was employed to calculate the TON_H2_.

Thus, the anchoring strategy used for the integration of **2** to MWNT allowed to outperform the simple physisorbtion of **1** at MWNT. Indeed, the pyrene moieties improving both surface loading and grafting stability, consequently improving both current densities and hydrogen production over time.

***Post operando*** **analysis**. In order to further understand the fate of the catalyst after long term electrolysis, *post operando* analysis were performed on the **2**/MWNT modified electrode using CV, UV‐Vis, FTIR and X‐ray photoelectron spectroscopy (XPS).

**Figure 6:**
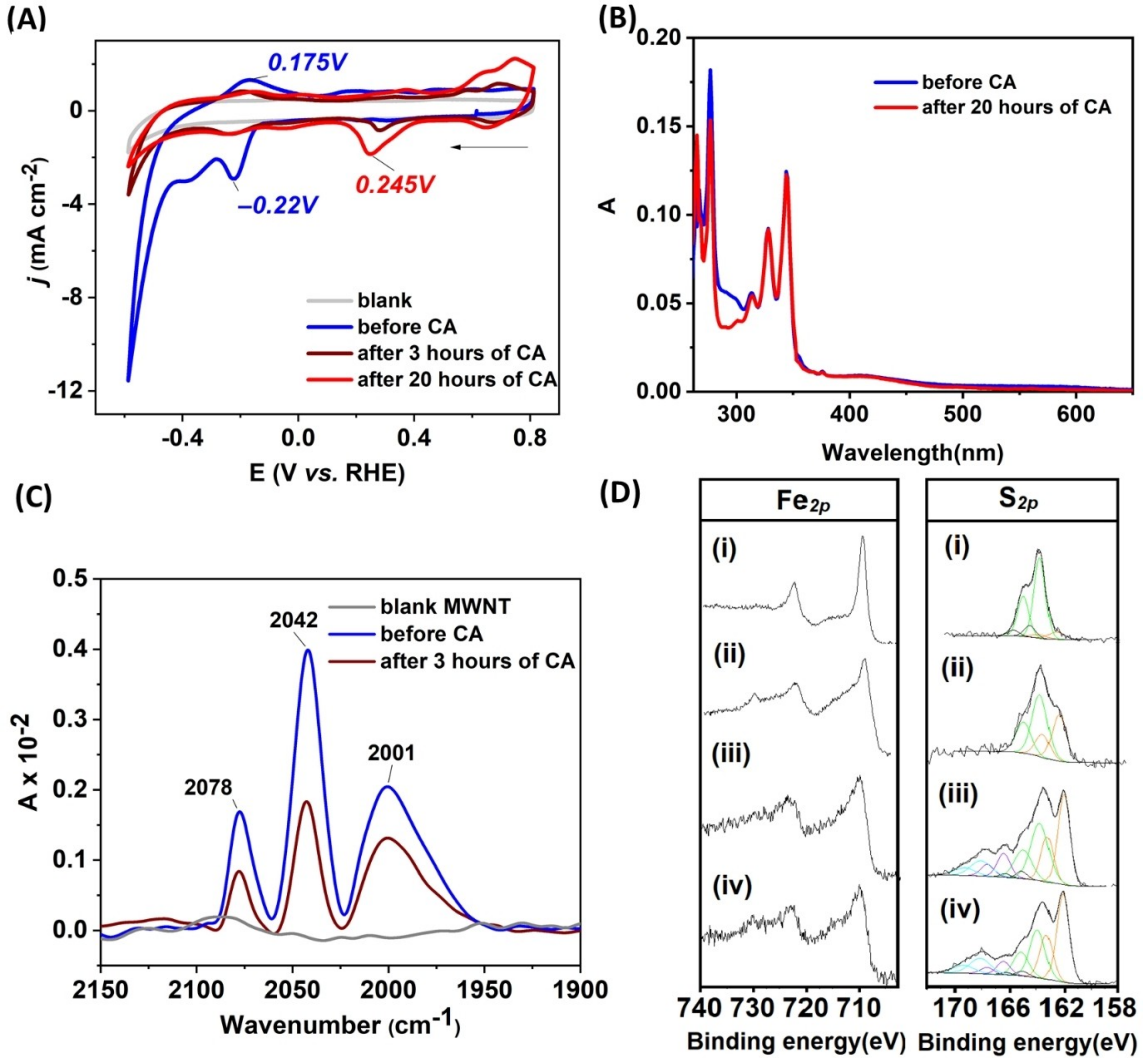
Stability of the **2**/MWNT functionalized electrodes during sustained H_2_ production monitored by CV and spectroscopic technique (CV and UV‐Vis, FTIR and XPS). (A) CV of complex **2**/MWNT before (blue trace), after 3 h (brown trace) and 20 h (red trace) of CA (ν=100 mV s^−1^); (B) UV‐Vis spectra of **2**/MWNT in 3 mL of DMF (by desorption of catalyst from film) before (blue trace) and after 20 h (red trace) of CA; (C) ATR‐ FTIR spectra of **2**/MWNT before (blue trace) and after 3 h (brown trace) of CA; (D) XPS showing binding energy correspond to 2p orbital of Fe and S for (i) **2** and **2**/MWNT (ii) before(iii) after 65 min and (iv) 175 min of CA with the potential poised at −0.49 V vs. RHE, in 0.2 M sodium phosphate buffer of pH 7.

After 3 h of electrolysis, CV scans showed a loss of catalytic current at E_red_=−0.49 V (from −4.64 to −1.35 mA cm^−2^) as well as non‐turnover signal response at E_red_=−0.22 V (from −2.9 to −1.0 mA cm^−2^), as expected from the current loss observed in the CA experiments (Figure 6A). CV traces recorded after 20 h of electrolysis, revealed an even further decrease of the observed currents (Figure 6A). Moreover, a new irreversible reduction peak at E_red_=0.245 V vs. RHE and two oxidation peaks at 0.63 V and 0.74 V were observed after sustained electrolysis, which grew significantly in amplitude over time (Figure 6A). The origin of these processes could not be attributed with certainty, but as it appeared together with a loss of catalytic current it is highly unlikely to reflect a catalytically relevant species. In comparison, **1**/MWNT showed similar loss of the non‐turnover signal at E_red_=−0.21 V (from −1.91 to −0.67 mA cm^−2^) and reached currents very close to the blank MWNT electrode (−0.46 mA cm^−2^), after 3 h. In parallel, the signal from the quinone/quinol redox event at E_red_=0.12 V showed a considerable drop (from −4.29 to −1.69 mA cm^−2^) during the first three hours and continued to decrease during the remaining 20 h (Figure S16). UV‐Vis spectra performed of the desorbed complex **2** from the modified MWNT film in DMF, revealed negligible intensity loss of absorption between 255–350 nm associated with the pyrene moieties (Figure 6B). ATR‐FTIR spectra of the **2**/MWNT functionalized electrode were acquired in the 2100 to 1600 cm^−1^ region and allowed to observe the characteristic signature of the CO groups. Compared to freshly prepared electrodes, the spectra showed between 35 % and 55 % loss of intensity of the characteristic CO peaks at 2001, 2042 and 2078 cm^−1^ after three hours of electrolysis (Figure 6C). Analogues loss of intensity of CO peaks was noticed during multiple cycles of CV as well (Figure S17). Thus, while the Fe‐CO core appears to degrade over the course of the electrolysis, the pyrene anchoring groups are retained at the surface of the electrode even after 20 h of electrolysis.

X‐ray photoelectron spectroscopy (XPS) experiments were performed to gain a precise elemental analysis of the film surfaces. XPS spectra of pure complex **2** displayed binding energies of 709.4 eV and 722.5 eV (Fe_*2p*_), as well as peaks at 163.6 eV (S_*2p*_), 285 eV (C_*1s*_ in C=CH), 286.5 eV (C_*1s*_ in C−O) and 532.1 eV (O_*1s*_) consistent with previous reports of analogous {(μ‐S_2_)Fe_2_(CO)_6_} derivatives (Figure 6D (i), S18). Spectra collected of **2**/MWNT, after 1 CV cycle in pH 7 buffer, confirmed similar binding energies for all elements except sulfur (S_*2p*_) where an additional peak centered at 162.1 eV (brown trace) emerged (Figure 6D spectrum (ii), and S19). This observation indicated the rapid formation of two different types of S_*2p*_ environments on the electrode. Subsequently, XPS data after 1 and 3 h of electrolysis revealed continuous decline of the signal at 709.4 eV and 722.5 eV hinting towards a loss of Fe whereas the signals for the other elements remained stable (Figure 6D (iii), (iv) and S19, & Table S4). Specifically, sulfur which binds the Fe_2_(CO)_6_ core to the rest the molecule attached on the electrode remained constant in term of relative ratio throughout the analysis (Table S4). Still, the shape of the peak assigned to S_*2p*_ displayed considerable changes with the appearance of a relatively intense peak centered at 162.1 eV and two additional peaks between 166 and 170 eV following electrolysis (Figure 6D). The peak centered at 162.1 eV was attributed to the formation of thiolate (RS^**−**^) via for example cleavage of one of the Fe−S bonds during cycling.[^73^, ^76^] The peak centered at 166–170 eV illustrated a gradual appearance of various sulfonate species (e. g. RSO_n_^**−**^ where n=2, 3) on the electrode surface during electrolysis.[^76^, ^77^] The latter observation further supports the notion of Fe−S bond cleavage.

**Scheme 2:**
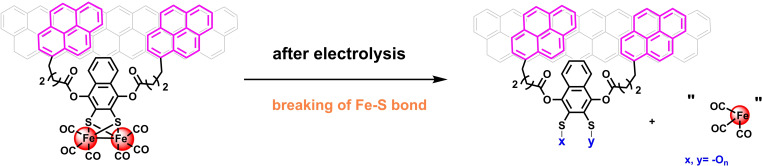
Possible degradation route of immobilized complex **2** during the electrocatalytic process.

In summary, the *post operando* analysis indicates that the loss of activity is the consequence of the degradation of the catalytic core, following the breaking of a Fe−S bond during reduction at low potential (cathodic) and electrolysis (Scheme 2). Lability of the Fe−S bonds agrees with mechanistic studies under homogenous conditions of similar diiron proton reduction catalysts i. e. [Fe_2_(μ‐bdt)(CO)_6_][^73^, ^78^] and [Fe_2_(μ‐S_2_C_3_H_6_)(CO)_6_],^[36]^ which have shown that one of Fe−S bonds breaks after two electrons reduction. This model is also consistent with the reported inactivation of a related diiron system, [Fe_2_(μ‐S‐C_6_H_4_‐p‐OH)_2_(CO)_6_], covalently attached on a silicon electrode reported recently.^[79]^

## Conclusion

We report the synthesis and extensive characterization of an original bioinspired [FeFe]‐H_2_ase mimic bearing two pyrene moieties, followed by its subsequent integration to MWNT‐based electrodes and an evaluation of its performance during electrocatalytic H_2_ production in aqueous media. Our data show that pyrene functionalization is efficient for the integration of such [FeFe]‐H_2_ase mimics at π*‐*conjugated surfaces. More specifically, it allowed the system to reach high surface loadings, ∼30 to 240 times higher than previously obtained through physisorption or covalent grafting. As a result, the grafted system displayed conservative TON_H2_ value of 4±2×10^3^ after 20 h of bulk electrolysis at −0.49 V vs. RHE in neutral aqueous conditions. The stability of the modified electrode was assessed using *post operando* characterization and showed that the loss of activity was attributable to the loss of iron carbonyl center, while the pyrene anchoring groups remained attached to the MWNT surface throughout long‐term electrolysis measurements. Hence, optimization of the molecular catalyst, together with their encapsulation in a suitable macromolecular matrix functionalized with anchoring groups such as pyrene could allow the development of efficient and robust bioinspired electrode materials for H_2_ production.

## Experimental Section

### Synthesis

Synthesis and characterization of [μ‐2,3‐(naphthalene‐1,4‐diylbis(4‐(pyren‐1‐yl)butanoate)‐dithiolato]bistricarbonyliron (**2**): In a dry, degassed Schlenk flask was loaded 156 mg (0.312 mmol) of (μ‐1,4‐naphthoquinone‐2,3‐dithiolato)Fe_2_S_2_(CO)_6_ (**1**, 100 % oxidized to naphthoquinone adduct as per ^1^H NMR)^[80]^ and 46.8 μL (0.748 mM) of NaCNBH_3_. The flask was then flushed three times with argon before the mixture was dissolved in 4.8 mL of dry, deaerated THF, and stirred for 2 h at room temperature. The resulting dark brown mixture was treated with 72.5 μL (0.42 mmol) of N,N‐Diisopropylethylamine at 10 °C and stirred for another 20 minutes. Subsequently, 300.6 mg (0.78 mmol) of 1‐pyrenebutyric acid N‐hydroxysuccinimide ester was added and the mixture stirred at room temperature for 14 h under argon. The resulting crude product was filtered through a small silica plug with CH_2_Cl_2_. The filtrate was concentrated under reduced pressure to about 500 μL and purified on a silica gel column with 20 % dichloromethane in hexane as an eluent to give **2** as an orange solid (150 mg, 46 %). The structure was further confirmed by X‐ray crystallography (Figure 1, S1). Recrystallization was performed in CH_2_Cl_2_, in which the product was dissolved at a concentration of 20–25 mg mL^−1^ and heated at 40 °C for 3–5 min followed by a slow evaporation at room temperature for 4 days to yield dark orange needle shaped single crystals.

^1^H NMR (350 MHz, CDCl_3_, 298 K) δ (ppm): 8.16–7.92 (9H, from pyrene ring, m), 7.5 (1H, from naphthalene group, dd *j*=6.42, 3.28 Hz,), 7.37 (2H, from naphthalene group, dd *j*=6.27, 3.15 Hz, ), 3.57 (2H, −OCCH_2_CH_2_C**H_2_** (C_16_H_9_), t *j*=7.53 Hz, ), 2.88 (2H, −OC**CH_2_** CH_2_CH_2_(C_16_H_9_), t *j*=7.38 Hz,), 2.43 (2H, −OCCH_2_C**H_2_** CH_2_(C_16_H_9_) m) (Figure S4).

^13^C NMR (350 MHz, CDCl_3_, 298 K) δ (ppm): 170.28 (−OC**C**H_2_CH_2_CH_2_(C_16_H_9_)), 135 (**C**−O, from naphthalene group), 131.5 (from to pyrene), 128–125 (from aromatic ring of naphthalene group and pyrene), 33.5 (−OCCH_2_CH_2_ **C**H_2_(C_16_H_9_)), 32.8 (−OC**C**H_2_CH_2_CH_2_(C_16_H_9_)), 26.6 (−OCH_2_ **C**H_2_CH_2_(C_16_H_9_)) (Figure S5).

FTIR (in CHCl_3_) v_max_ (cm^−1^): 2081, 2049 and 2010 (CO ligated to Fe), 1769 (−C=O of ester bond) (Figure 2A).

UV‐Vis (in DMF) λ, nm (*ϵ*, mol^−1^ cm^−1^): 255–350 (*ϵ*_345nm_=1.95×10^5^), 371–435 (*ϵ*_390nm_=1.76×10^4^), 435–540 (*ϵ*_475nm_=3.08×10^3^) (Figure 2B).

HRMS (ESI) m/z calculated for C_56_H_35_Fe_2_O_10_S_2_: 1042.0292; found: 1043.0365 [M+H] ^+^ (Figure S1).

**Preparation of the [FeFe]/MWNT electrode**: The MWNT electrodes were prepared by drop casting 20 μl of a MWNT suspension in ethanol (3 mg mL^−1^) on a glassy carbon electrode (d=1.6 mm) followed by drying for 30 min at room temperature until the film was dried. Then, the MWNT electrode was soaked in a catalyst solution in DMF (3.6 mM) for 10 min before being successively gently rinsed with DMF and water in order to remove unbound or weakly bound catalyst and DMF, respectively. For the preparation of the electrodes for heterogeneous electrochemistry in acetonitrile, the rinsing with water was avoided. The resulting geometrical electroactive surface was estimated to approximately 0.2 cm^2^ (Figure S9).

**Electrochemistry**: All electrochemical analysis were performed on a Biologic SP‐300 workstation at room temperature (298 K). A platinum coil was used as counter electrode and an Ag/AgCl (in 3 M KCl) electrode was used as reference. The reference electrode was calibrated before each experiment as per previous report.^[81]^ The potentials are reported against Ferrocene/Ferrocenium (Fc^+/0^) redox potential in organic media and against reversible hydrogen electrode potential (RHE) in aqueous media. A glassy carbon electrode (d=1.6 mm) was used as working electrode (unmodified for homogenous condition). Cyclic voltammetry experiments were performed under an argon atmosphere in a 3 mL electrochemical cell. The cell was purged for 20 min at 5 mL min^−1^ rate before analysis to degas the solvent. The analysis in organic solvent (DMF, CH_3_CN) was performed with 0.1 M TBAPF_6_ as supporting electrolyte. In aqueous media experiments were performed in 0.2 M phosphate buffer at pH 7.

**Chronoamperometry measurments coupled with gas chromatography**: Electrolysis were performed in a five‐neck glass vessel (volume of working electrode compartment was 55 mL including the headspace) as electrochemical cell. Each neck was sealed with rubber septum to avoid leakage. The cell was separated into two compartments by a glass frit in order to limit oxygen diffusion from the anodic to the cathodic compartment during electrolysis. The cell was deaerated for 20 min with argon before analysis. The cathodic part of the cell was coupled with gas chromatography with continuous argon flow with rate of 5 mL min^−1^ to quantify H_2_ produced during analysis. The data for gas chromatography was collected at every 1 min interval.

**CCDC data**: 2183292 (for **1**)) contains the supplementary crystallographic data for this paper. These data are provided free of charge by the joint Cambridge Crystallographic Data Centre and Fachinformationszentrum Karlsruhe Access Structures service.

## Conflict of interest

The authors declare no conflict of interest.

1

## Supporting information

As a service to our authors and readers, this journal provides supporting information supplied by the authors. Such materials are peer reviewed and may be re‐organized for online delivery, but are not copy‐edited or typeset. Technical support issues arising from supporting information (other than missing files) should be addressed to the authors.

Supporting Information

## Data Availability

The data that support the findings of this study are available from the corresponding author upon reasonable request.
