# Supplementary material for: Non‐Covalent Integration of a [FeFe]‐Hydrogenase Mimic to Multiwalled Carbon Nanotubes for Electrocatalytic Hydrogen Evolution
Source: Chemistry. 2022 Oct 19;28(69):e202202260. doi: 10.1002/chem.202202260 (PMC10092503; doi:10.1002/chem.202202260)
Supplement: Supplementary file 1 — Supporting Information [file CHEM-28-0-s001.pdf]

Chemistry–A European Journal

Supporting Information

Non-Covalent Integration of a [FeFe]-Hydrogenase Mimic to Multiwalled Carbon Nanotubes for Electrocatalytic Hydrogen Evolution

Afridi Zamader, Bertrand Reuillard,* Jacques Pécaut, Laurent Billon, Antoine Bousquet, Gustav Berggren,* and Vincent Artero

Supporting information

Experimental section 2
General 2
X-ray diffraction 2
X-Ray Photoelectron Spectroscopy 3
Sample preparation for post operando XPS and ATR-FTIR characterization 3
Desorption of complex 2 post operando for UV-Vis measurements 4
Calculation 4
Calibration of potential 4
Calculation of surface coverage by catalyst 4
Calculation of Turnover number (TON) and turnover frequency (TOF) 5
Supporting Figures and Tables 5
References 25

Experimental section

General

All chemicals were purchased from Sigma-Aldrich and used as received unless otherwise stated. Multiwall carbon nanotubes (MWNT) powder was purchased from NANOCYL® NC7000™ series. The solvents used for synthesis were distilled and deaerated through argon purging before each step of reaction. The organic solvents used in electrochemical analysis, 99.8% anhydrous dimethylformamide and acetonitrile (extra dry over molecular sieves and stored in Argon) were purchased from Sigma-Aldrich and ACROS organic respectively. The Milli-Q water used for preparing buffer was purified through Millipore system.

All synthetic steps were performed using Schlenk techniques. Purification were done using column chromatography. NMR (^{13}C and ^{1}H) were performed with a Bruker AVANCE III-300 MHz spectrometer. UV-Vis spectra were recorded on a Shimadzu UV-1800 spectrometer and fluorescence spectra were performed on JASCO FP-6500 fluorescence spectrometer. IR spectra were recorded on a PerkinElmer Spectrum spectrometer. The high-resolution mass spectrometry and X-ray crystallography were analyzed on maXis II bruker instrument equipped with an electrospray source (positive mode) and Xcalibur and Sapphire3 diffractometer respectively, at the Service des Systèmes Moléculaires et nanoMatériaux pour l'Énergie et la Santé of the CEA Grenoble. The glassy carbon electrodes were polished with 1µM diamond paste using Struers LaboPol-1 polishing machine. The H_2 gas was analyzed on Micro Gas Chromatograph S3000 (SRA Instruments) with a diamond LV Ms5A 14m module, operated using the Soprane chrome interface. The flow of the feeding gas (Argon) was controlled using Bronkhorst EL-FLOW mass-flow meters with flowrate of 5 mL min^{-1}. The X-ray photo electron spectroscopy (XPS) was performed on Escalab 250 Xi spectrometer using a monochromatized Al Kα radiation (hv = 1486.6 eV) at liquid nitrogen temperature. Complex **1** or [µ-2,3-(1,4-naphthoquinone)-dithiolato]bistricarbonyliron was synthesized and characterized using previously reported protocol (Scheme 1).[1]

X-ray diffraction

Data was collected at the X-ray Diffraction Facility of CEA Grenoble. Single translucent intense orange needle-shaped crystals of complex **2** were obtained by recrystallization from DCM with co-crystallization of two solvent molecule per one active site. A suitable crystal of 0.45 x 0.12 x 0.07 mm^3 was selected and mounted on a suitable support on an Xcalibur, Sapphire3 diffractometer. The crystal was kept at a steady T = 150.2(3) K during data collection. The structure was solved with the ShelXS

n/a[2] structure solution program using Patterson method, solution method and by using Olex2[3] as the graphical interface. The model was refined with version 2018/3 of ShelXL 2018/3[4] using Least Squares minimization. Crystallographic figures were obtained using Mercury (Version 3.9). The asymmetric unit contains one complex and two DCM molecules. First DCM molecule contains two different positions for one Cl atom, and the second DCM molecule is highly disordered (Cl atom are dispersed on five positions and they count for 2 atoms). Some selected parameters (atomic coordinates, bond lengths, bond angles and torsion angles) of the obtained crystal were mentioned in Table S6–S10. Full supplementary crystallographic data can be found in CCDC 2183292. <url href="https://www.ccdc.cam.ac.uk/services/structures?id=doi:10.1002/chem.20220XXX"> 2183292 (for 1))</url> contains the supplementary crystallographic data for this paper. These data are provided free of charge by the joint Cambridge Crystallographic Data Centre and Fachinformationszentrum Karlsruhe <url href=" http://www.ccdc.cam.ac.uk/structures ">Access Structures service</url>.

X-Ray Photoelectron Spectroscopy

XPS measurements were carried out with a THERMO Escalab 250Xi spectrometer, using focused monochromatic Al Kα radiation (hν = 1486.6 eV). The XPS spectra were recorded with constant pass energy of 20 eV. Charge neutralization was used for all the acquisitions. The pressure in the analysis chamber was around 5.10−7 mbar. Short acquisition time spectra were recorded before each experiment to check that the samples didn't suffer degradation during the X-ray irradiation. The binding energy scale was calibrated using the C_{1s} peak at 285.0 eV from the hydrocarbon contamination always present at the samples surface. The curves fit for core peaks were obtained using a minimum number of components. For orbitals with l≠0, the spectrum appears under doublet shape, due to the spin-orbit coupling. For example, the S_{2p} spectrum corresponding to one kind of sulfur atoms environment appears under the form of two peaks named $S2p_{3/2}$ and $S2p_{1/2}$. It was further validated by the area ratio, the FWHM (full width at half maximum) ratio and the energy separation that are well known and fixed for each $S2p_{3/2}$ - $S2p_{1/2}$ coupled peaks as per "NIST X-ray Photoelectron Spectroscopy Database". The quantification and curve fitting were performed using CASA XPS software.

Sample preparation for post operando XPS and ATR-FTIR characterization

A larger glassy carbon disk (A = 1 cm^2) was used to prepare the working electrode following the same procedure as discussed earlier except 100 µl of MWNT suspension used here. For XPS, the catalyst functionalized MWNT modified glassy carbon disk electrode was gently rinsed with water, dried in air followed by directly set up in XPS machine to perform elemental analysis. For ATR-FTIR, catalyst

functionalized MWNT film was detached carefully from glassy carbon electrode surface and directly analyzed using infrared spectroscopy via ATR mode.

Desorption of complex 2 post operando for UV-Vis measurements

The immobilized catalysts were extracted from electrode by detaching the catalyst functionalized CNT film followed by gently shaking in 3 mL DMF solution for 10–15 minutes. The resulted suspension was filtered to get rid of MWNT and then the filtrate was used to record UV-Vis spectra.

Calculation

Calibration of potential

E (*vs.* RHE) = E (*vs.* Ag/AgCl; 3M KCl) + E_0 (Ag/AgCl; 3M KCl) + 0.059 x pH ……… (1)

E (*vs.* SHE) = E (*vs.* Ag/AgCl; 3M KCl) + E_0 (Ag/AgCl; 3M KCl) ……….(2)

E (*vs.* $Fc^{+1/0}$) = E (*vs.* Ag/AgCl; 3M KCl) – E ($Fc^{+1/0}$ *vs.* Ag/AgCl/KCl) ……….(3)

Where,

- Where, E_0 (Ag/AgCl; 3 M KCl) = 0.21 V *vs.* SHE at room temperature.
- E_0 ($Fc^{+1/0}$ *vs.* Ag/AgCl; 3M KCl) = Equilibrium potential ($E_{1/2}$) for Ferrocenium/Ferrocene redox event against Ag/AgCl; 3M KCl electrode in respective organic solvents.

Calculation of surface coverage by catalyst

(a) From cyclic voltammetry,

$$\Gamma_{\mathrm{CV}} = \frac{\mathrm{Q}}{\mathrm{n}\ x\ \mathrm{F}\ x\ \mathrm{A_{electrode}}} \qquad \dots\dots\dots\dots\dots\dots (4)$$

Where,

Γ is the surface loading (mol cm^{-2}), Q is the charge (C) obtained by integration of the oxidation wave of the corresponding redox event. n is the number of electrons involved in the process (= 1), F is Faraday constant (= 96485 C mol^{-1}) and $A_{electrode}$ is the surface of electrode (= 0.2 cm^2).

(b) From UV-Vis spectroscopy,

$$\Gamma_{UV} = \frac{\mathrm{A_{345nm}}\ \mathrm{x}\ \mathrm{V}}{\varepsilon_{345\mathrm{nm}}\ x\ \mathrm{l}\ \ x\ \mathrm{A_{electrode}}\ x\ 1000} \qquad \dots\dots\dots\dots\dots. (5)$$

Where, $A_{345\ nm}$ is absorbance at 345 nm, ε_{345nm} is extinction coefficient at 345 nm (= 1.95 x 10^5 M^{-1} cm^{-1}). V is volume of solvent used to desorb catalysts from electrode (= 3 mL). l is path length of cuvette (=1 cm). $A_{electrode}$ is surface area of electrode (= 0.2 cm^2).

Calculation of Turnover number (TON) and turnover frequency (TOF)

$$n_{H2} \text{ accumulated per cm}^2 = \frac{n_{H2} \text{ accumulated by } \mathbf{2}/\text{MWNT} - n_{H2} \text{ accumulated by MWNT}}{\text{A}_{\text{electrode}}} \quad \ldots\ldots\ldots\ldots\ldots\ldots (6)$$

$$\text{TON} = \frac{\text{nH}_2 \text{ accumulated per cm}^2}{\Gamma} \quad \ldots\ \ldots.\ldots\ \ldots..(7)$$

Where, nH_2 = mol of H_2 (mol), Γ is the surface loading (mol cm^{-2}).

Supporting Figures and Tables

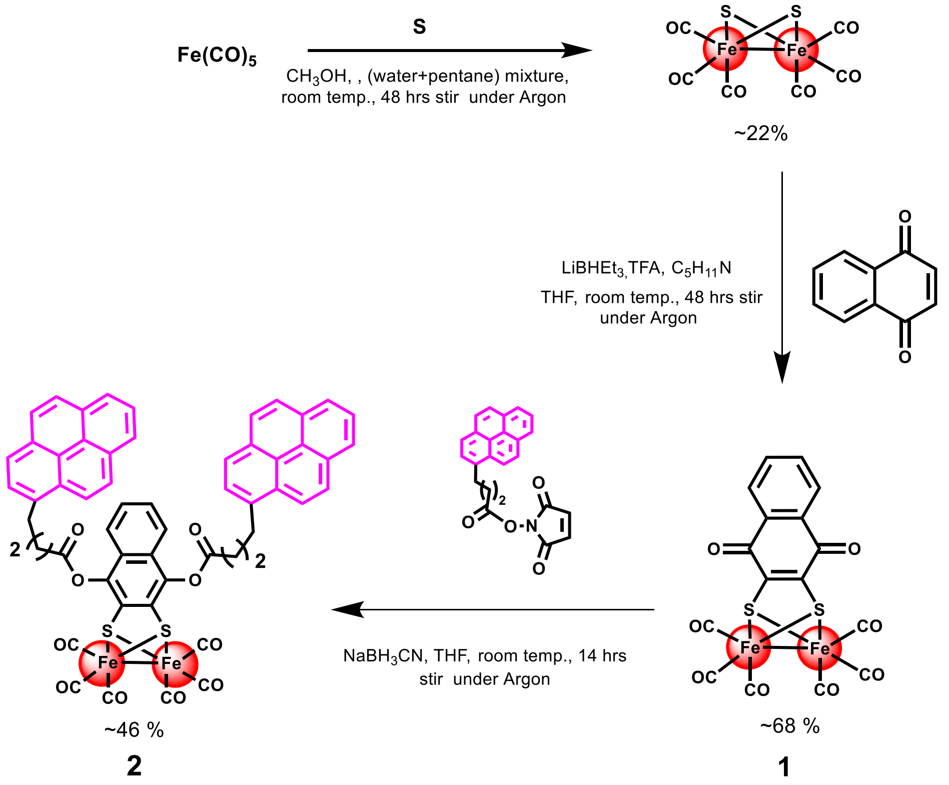

Scheme S1: Synthetic route towards complex **1** and **2**.

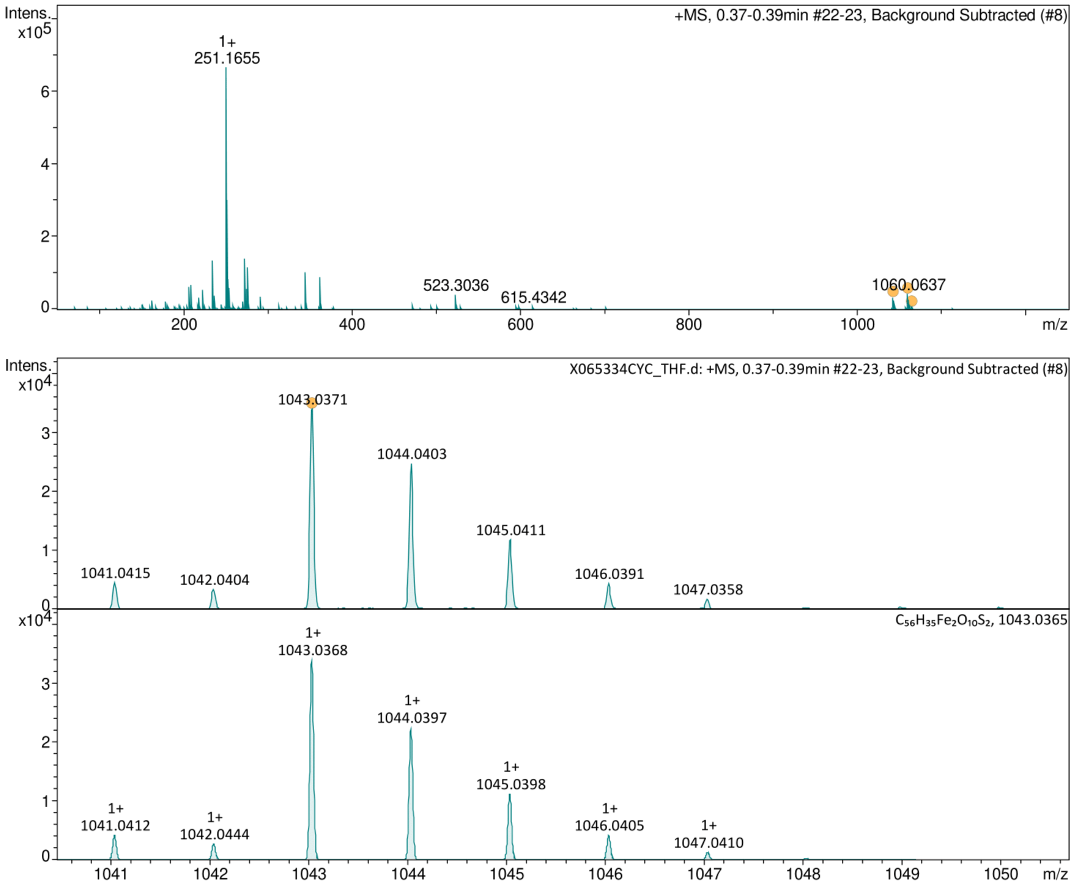

Figure S1: High resolution mass spectrometry (HRMS) of complex **2** in THF. Calculated for $C_{56}H_{35}Fe_2O_{10}S_2$: 1042.0292; found: 1043.0365 [M + H]$^+$, 1060.063738 [M + NH$_3$]$^+$.

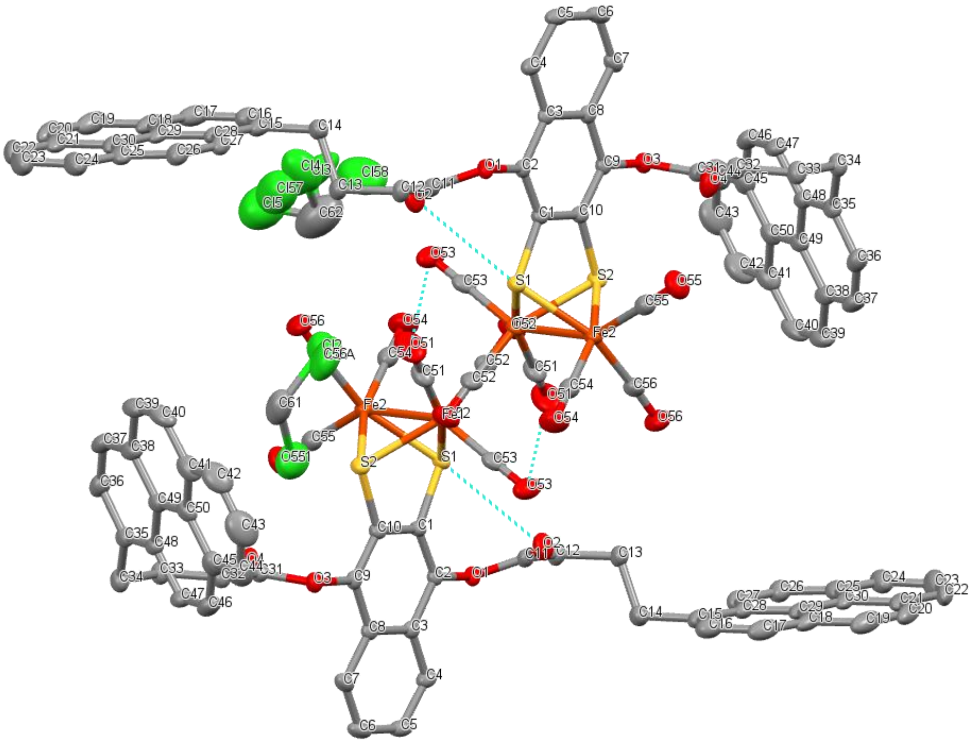

Figure S2: X-ray crystallography of complex **2**. (hydrogen atom omitted) showing intra and inter molecular interaction to form a caged shaped structure. Fe, S, C and O atoms are represented by orange, yellow, grey and red colours, respectively. Cl (green) atoms stem from co-crystallisation of CH_2Cl_2 used for recrystallization. Selected bond distance (Å): Fe1–Fe2, 2.4802(5); Fe1–S1, 2.2598(8); Fe1–S2, 2.2776(8); Fe1–C51, 1.791(3); Fe1–C52, 1.789(3); Fe1–C53, 1.805(3); Fe2–S1, 2.2678(7); Fe2–S2, 2.2743(7); Fe2–C54, 1.793(3); Fe2–C55, 1.806(3); Fe1–C56, 1.796(3); S1-O2, 3.1897(1); O51–O53(1-x, -y, 1-z), 3.0145(1); mean plane pyrene (1-x, -y, 1-z)–C39, 6.9307(2); mean plane pyrene(1-x, -y, 1-z)–C40, 6.9472(2).

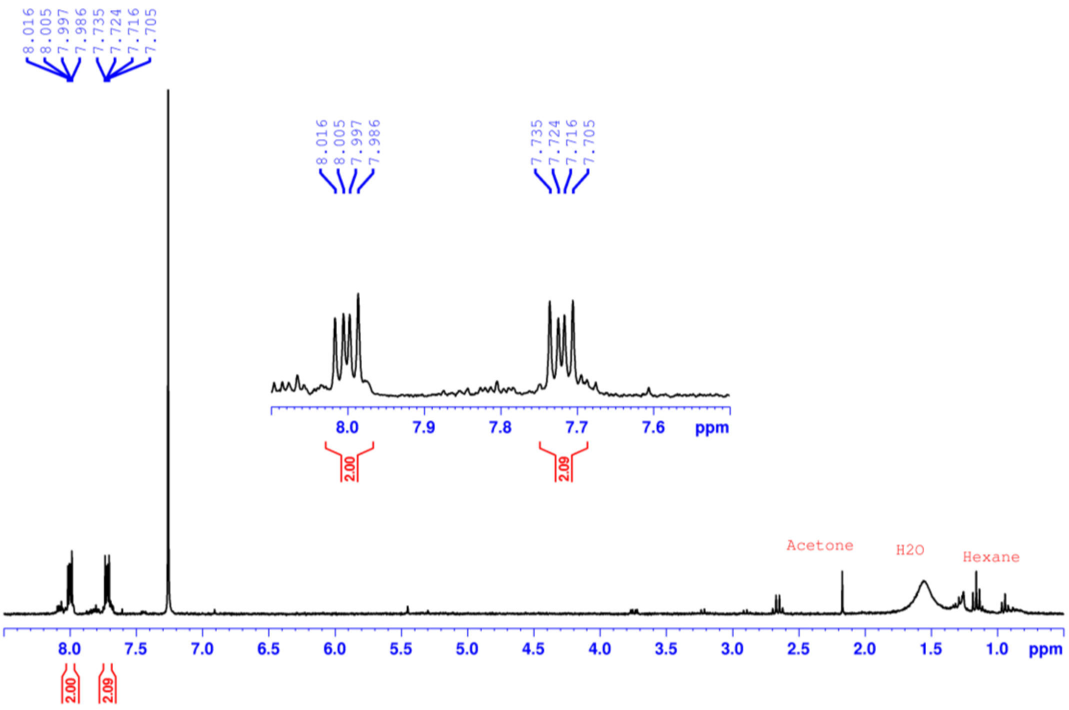

Figure S3 : ^{1}H NMR of complex **1** in $CDCl_3$.

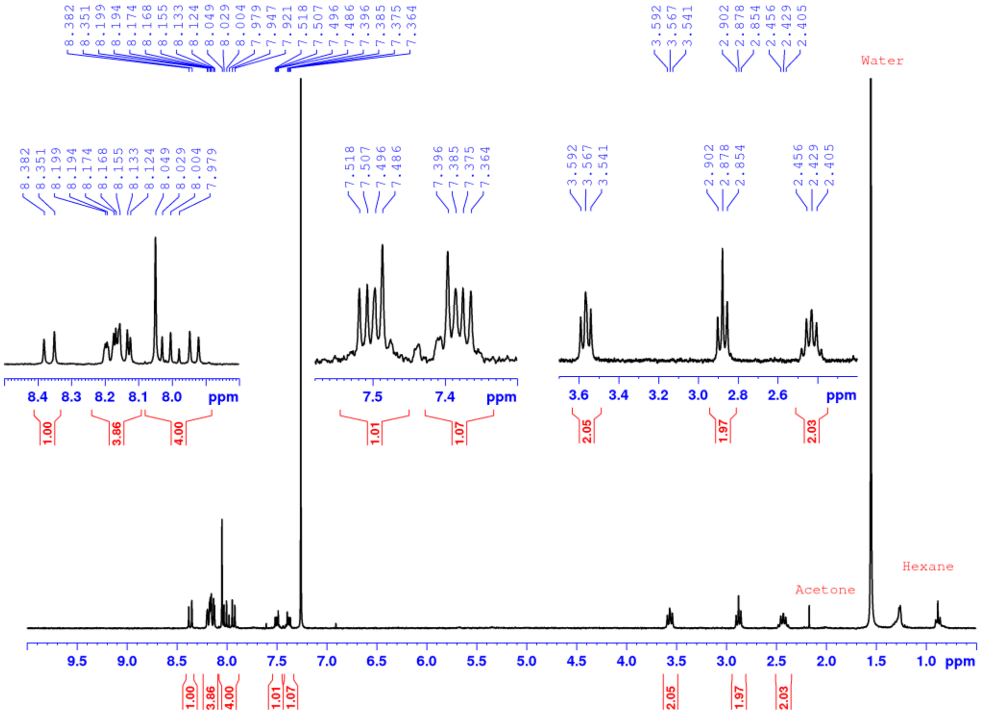

Figure S4: ^{1}H NMR of complex **2** in $CDCl_3$.

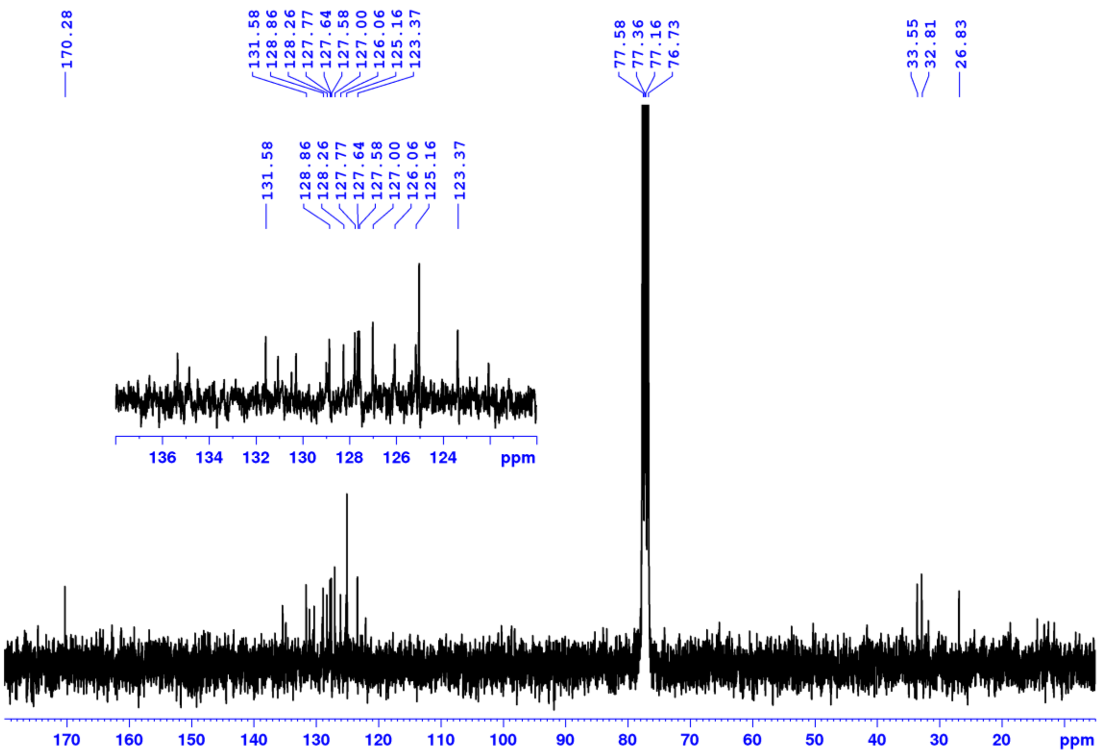

Figure S5: ^{13}C NMR of complex **2** in $CDCl_3$.

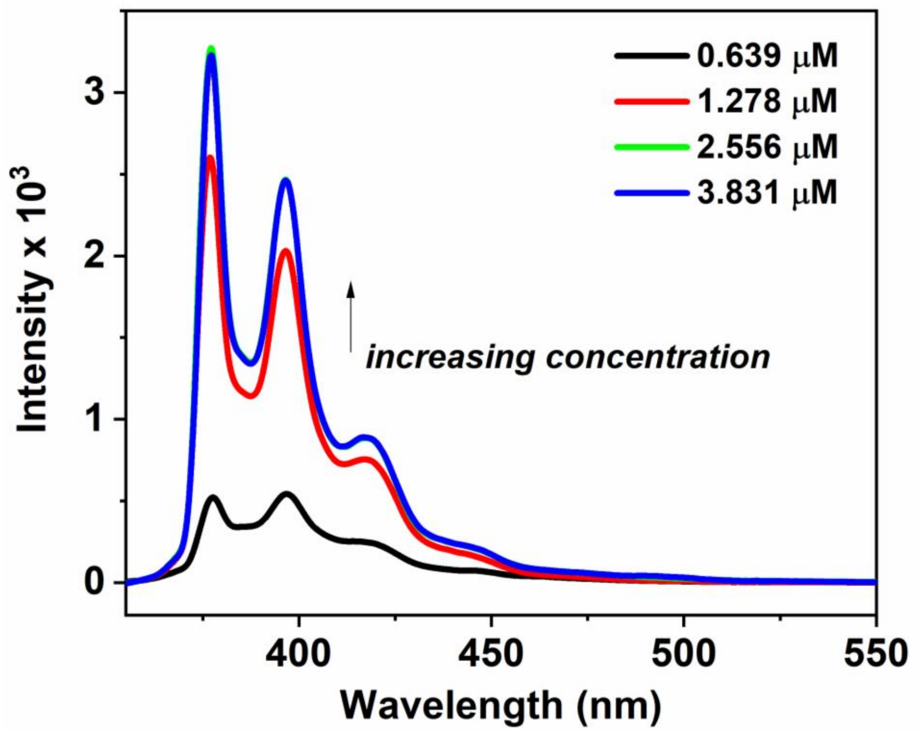

Figure S6: Fluorescence spectra of complex **2** in DMF, excitation wavelength of 314 nm.

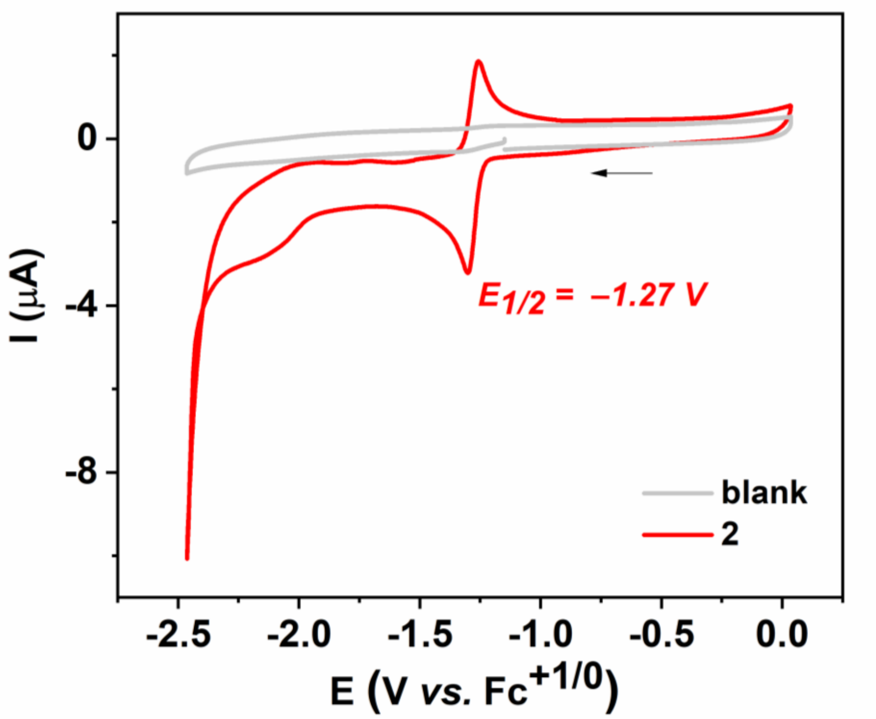

Figure S7: CV of complex **2** (~0.25–0.4 mM) solution of CH_3CN at room temperature. (ν = 100 mV s^{-1}, [$TBAPF_6$] = 0.1M).

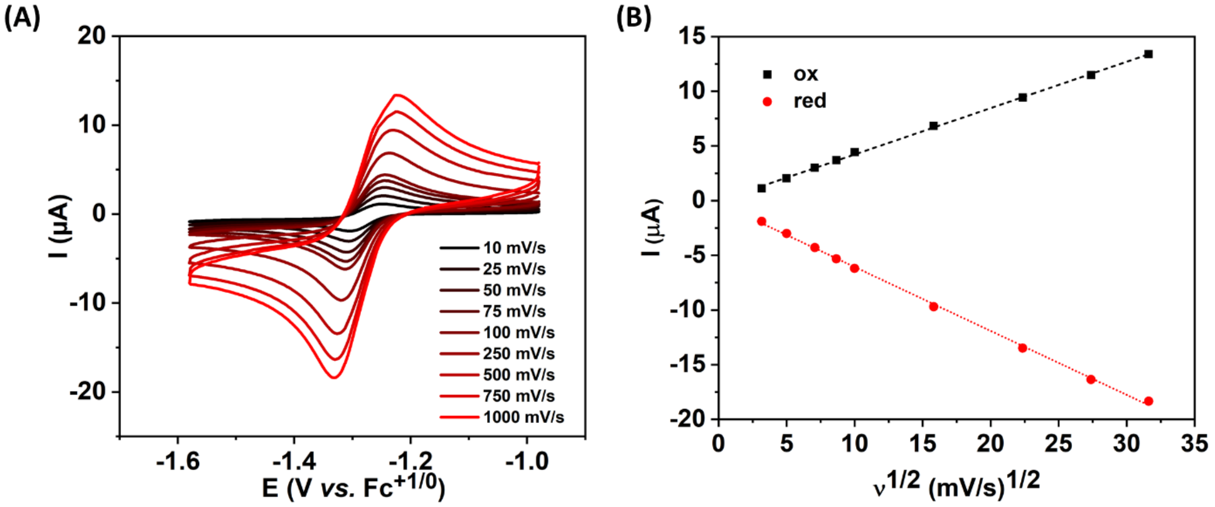

Figure S8: Scan rate dependence cyclic voltammetry studies of 1 mM of complex **2** in DMF at room temperature, [$TBAPF_6$] = 0.1 M. (A) CV at different scan rate (10–1000 mV s^{-1}) and (B) evolution of the redox peak current (I) with the square root of the scan rate ($\nu^{1/2}$).

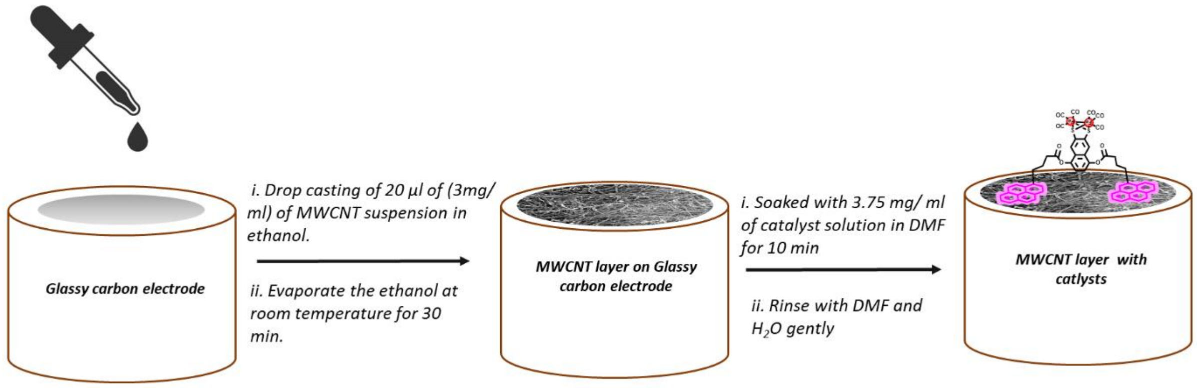

Figure S9: Schematic diagram of the preparation of catalyst functionalized MWNT film.

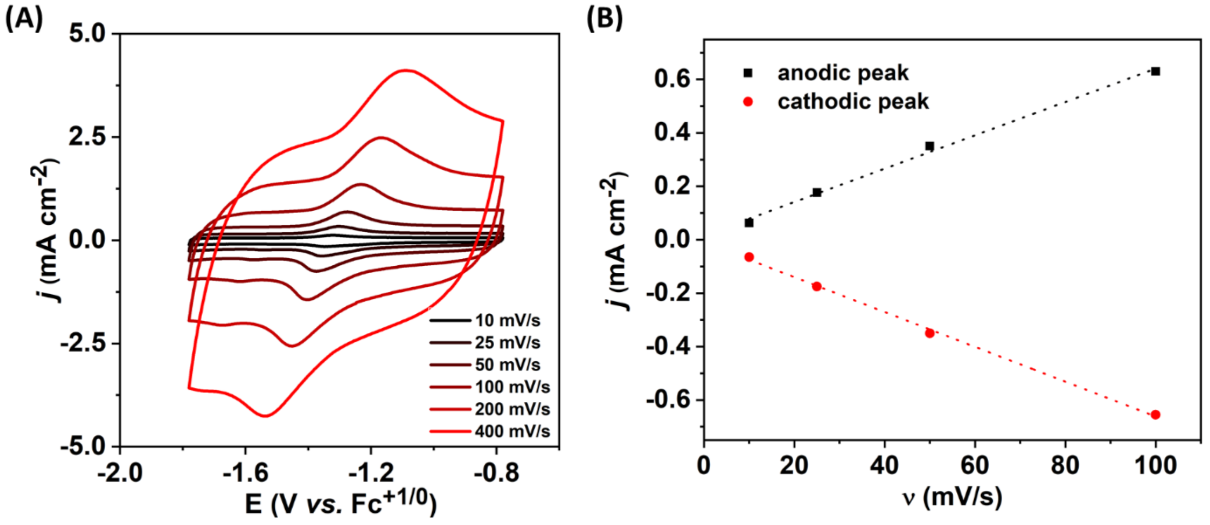

Figure S10: Scan rate dependence cyclic voltammetry studies of **2**/MWNT in CH_3CN at room temperature, [$TBAPF_6$] = 0.1 M. (A) at scan rate between 10–400 mV s^{-1} and (B) evolution of the redox peak current density (*j*) *vs*. scan rate (ν).

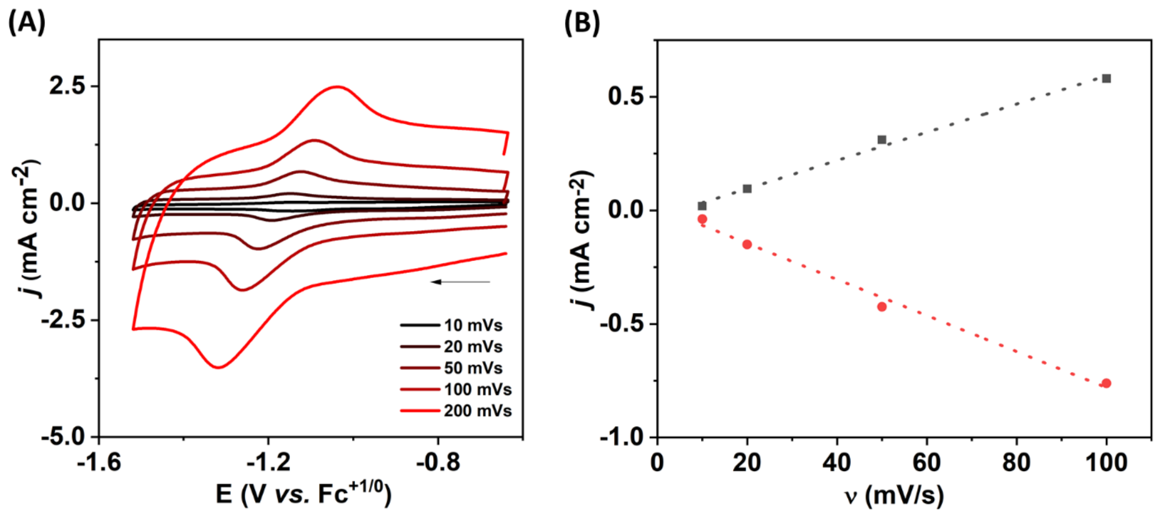

Figure S11: Scan rate dependence cyclic voltammetry studies of **1**/MWNT in CH_3CN at room temperature, [$TBAPF_6$] = 0.1 M. (A) at scan rate between 10–200 mV s^{-1} and (B) evolution of the redox peak current density (j) vs. scan rate (ν).

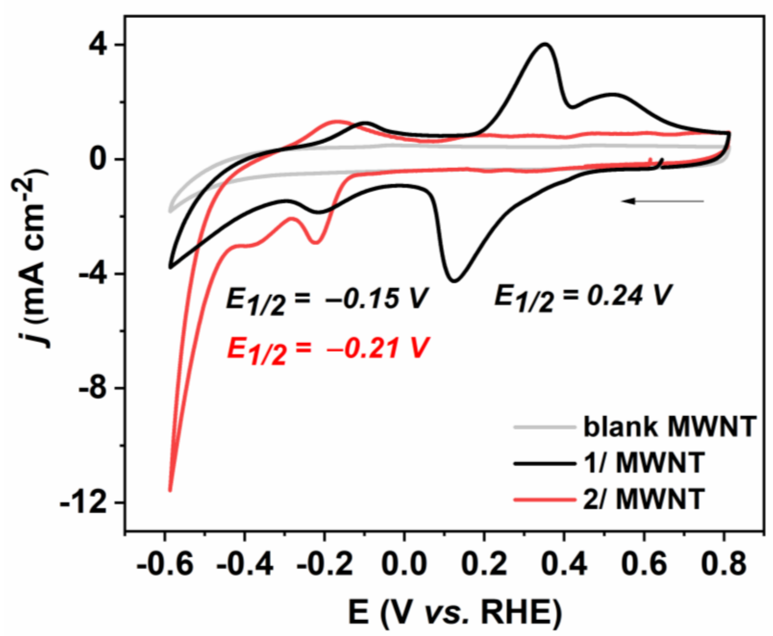

Figure S12: CV traces of the immobilized catalysts, **1**/MWNT (black trace) and **2**/MWNT (red trace), in 0.2 M sodium phosphate buffer of pH 7 at room temperature (ν = 100 mV s^{-1}). **1**/MWNT and **2**/MWNT displayed a quasi-reversible redox process at $E_{1/2}$ = –0.15 V and –0.21 V *vs.* RHE (ΔE_p = 120 mV), which could possibly be assigned diiron subsite based respectively. In addition, **1**/MWNT showed an additional reversible feature at 0.24 V *vs.* RHE due to quinone/quinol (2e⁻ and 2H⁺) redox event.

Table S1: Electrochemical analysis of Complex 2 at different conditions.

Catalyst	Type of CV	Solvent	$E_{1/2}$ for $Fe^{I}Fe^{I}/Fe^{0}Fe^{0}$
**2**	Homogeneous	0.1M $TBAPF_6$ in DMF	−1.26 V *vs.* $Fc^{+1/0}$ (quasi-rev.)<br>ΔE_p = 60 mV
		0.1M $TBAPF_6$ in CH_3CN*	−1.27 V *vs.* $Fc^{+1/0}$ (quasi-rev.)<br>ΔE_p = 45 mV
	Heterogeneous	0.1M $TBAPF_6$ in CH_3CN	−1.32 V *vs.* $Fc^{+1/0}$ (quasi-rev.)<br>ΔE_p = 170 mV
		0.2M sodium phosphate buffer of pH 7	−0.21 V *vs.* RHE (quasi-rev.)<br>ΔE_p = 48 mV**

(rev. = reversible, * partially soluble, conc. of catalyst ~0.25–0.4 mM, **this process was attributed to $Fe^{I}Fe^{I}/Fe^{I}Fe^{0}$ redox event)

Table S2: Loading of catalysts on electrode surface (see calculation details in section 1.2.)

**Catalyst**	$A_{345\ nm}$ after desorption of functionalised electrode (0.2 cm^2) in 3 mL DMF	Active sites loaded as per UV-Vis spectra (nmol cm^{-2})	Active sites loaded as per Electrochemistry* (nmol cm^{-2})
**2**	0.123 ± 0.001	9.5 ± 0.1	7.6 ± 0.9

*CV peak integration of anodic wave at $E_{1/2}$ = −1.32 V *vs.* $Fc^{+1/0}$ for **2**/MWNT (0.2 cm^2) in acetonitrile, [$TBAPF_6$] =0.1M.

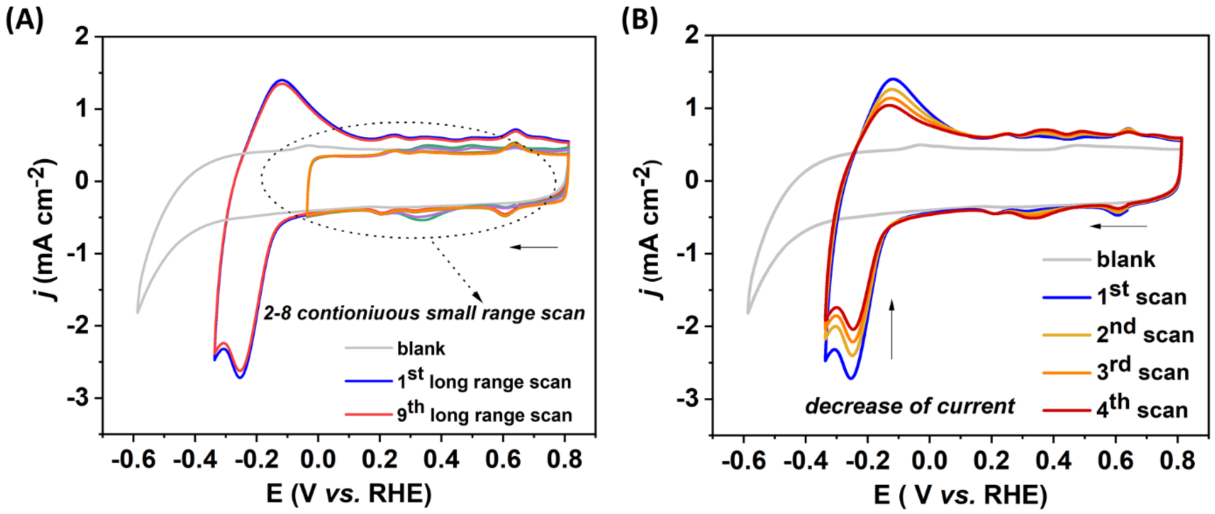

Figure S13: Effect of repeated scans on non-turn over current for **2**/MWNT at aqueous media (0.2 M sodium phosphate buffer, pH 7) at different potential windows at room temperature. (A) from 0.8 V to –0.03 V *vs.* RHE for the 7 repetitive scans in between for 1st and 9th scan from 0.8 V to – 0.33 V *vs.* RHE (B) that from 0.8 V to – 0.33 V *vs.* RHE in 0.2 M sodium phosphate buffer of pH 7 at room temperature (ν = 100 mV s^{-1}).

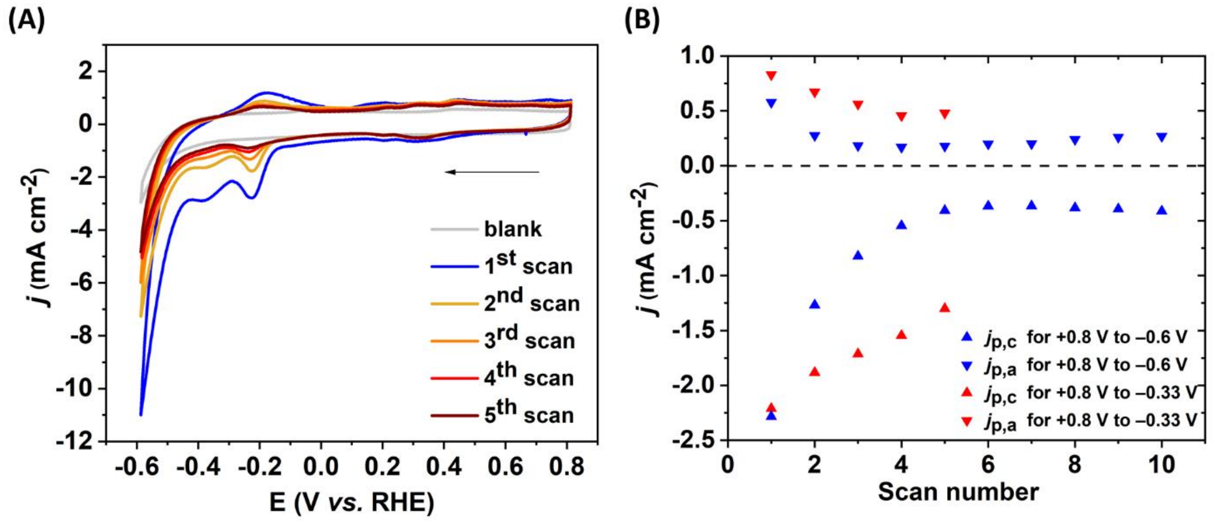

Figure S14: Effect of repeated scans on the non-turnover current of CV for **2**/MWNT in aqueous media (0.2 M sodium phosphate buffer of pH 7, ν = 100 mV s^{-1}) at room temperature. (A) CV of **2** /MWNT after repeated cycles (only first five cycles were shown where maximum current loss was observed) and (B) current loss during scans at different potential windows from + 0.8 V to –0.6 V *vs*. RHE (blue traces) and +0.8 V to –0.33 V *vs*. RHE (red traces). Here, $j_{p,c}$, $j_{p,a}$ are cathodic and anodic peak current density at –0.22 V *vs.* RHE and – 0.18 V *vs.* RHE respectively. Peak current density at plot bare background subtracted.

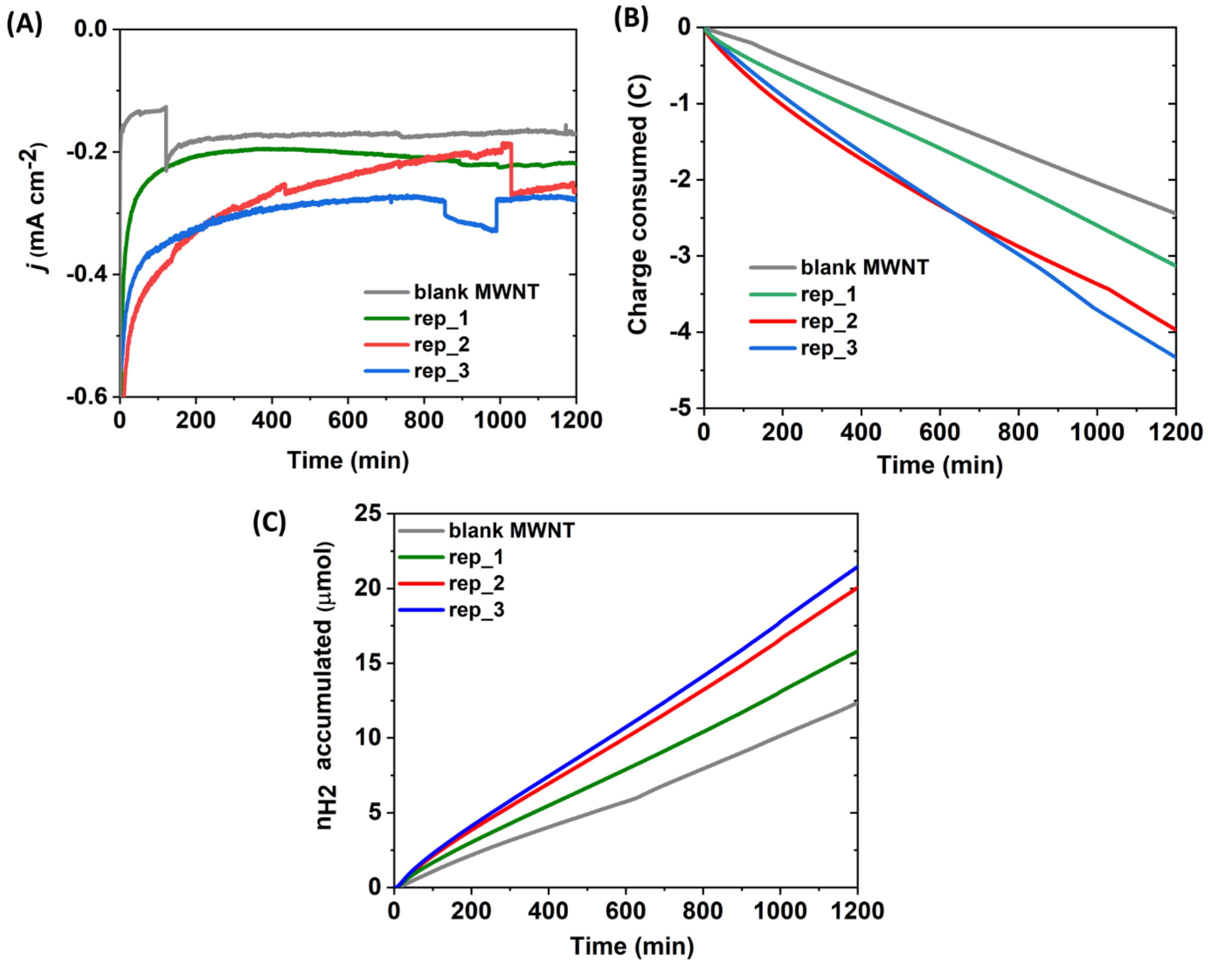

Figure S15: Replicates of the sustained H_2 production experiments ("rep_3" also shown in Figure 5 main text). The chronoamperometry traces (A) and corresponding evolution of H2 (B) and consumption of charge (C) recorded for three separate **2**/MWNT films (green, red and blue traces) and bare MWNT (grey trace) electrodes poised at –0.49 V *vs.* RHE in 0.2 M sodium phosphate buffer of pH 7 under argon at room temperature. The sharp fluctuation of currents between 900 to 1000 min in CA was due to bubble formation at the working electrode during electrolysis.

Table S3: Quantitative analysis from chronoamperometry. *j* = current density, TON = turnover number, FE = Faradic efficiency. TON_{H2} were calculated using by maximum value of loading (Γ_{UV}) to show the least value of it (see section S1.3 for details).

	***j* at starting after 2th min of CA (mA cm^{-2})**	***j* at the end of CA (mA cm^{-2})**	**Duration of CA (min)**	**Amount of H_2 produced (µmol)**	**Amount of charge consumed (C)**	**% FE**	**% loss of current during CA**	**TON**
Blank	−0.185	−0.17	1200	12.67	2.45	99.96	8	
**1**	−0.42 ± 0.9	−0.18 ± 0.01	1200	14.83 ± 0.6	2.89 ± 0.11	98.6 ± 0.4	58 ± 4	2 ± 1 x 10^3
**2**	− 0.71 ± 0.17	−0.25 ± 0.03	1200	19.3 ± 3	3.76 ± 0.6	99.1 ± 0.4	64 ± 4	4 ± 2 x 10^3

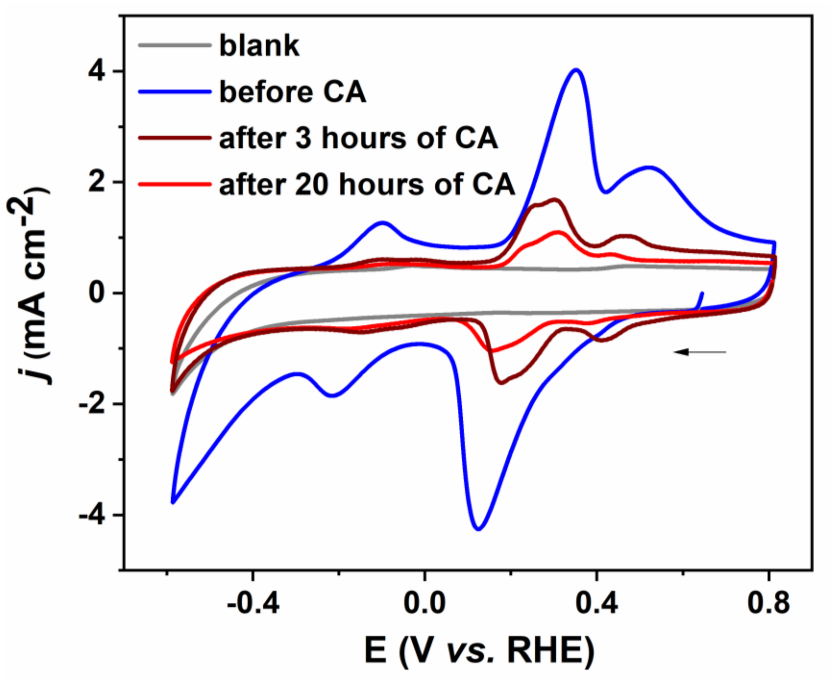

Figure S16: CV of complex **1**/MWNT before (blue trace), after 3 hours (brown trace) and 20 hours (red trace) of CA (ν = 100 mV s^{-1}). CA was performed with potential poised at −0.49 V *vs.* RHE, in 0.2 M sodium phosphate buffer of pH 7.

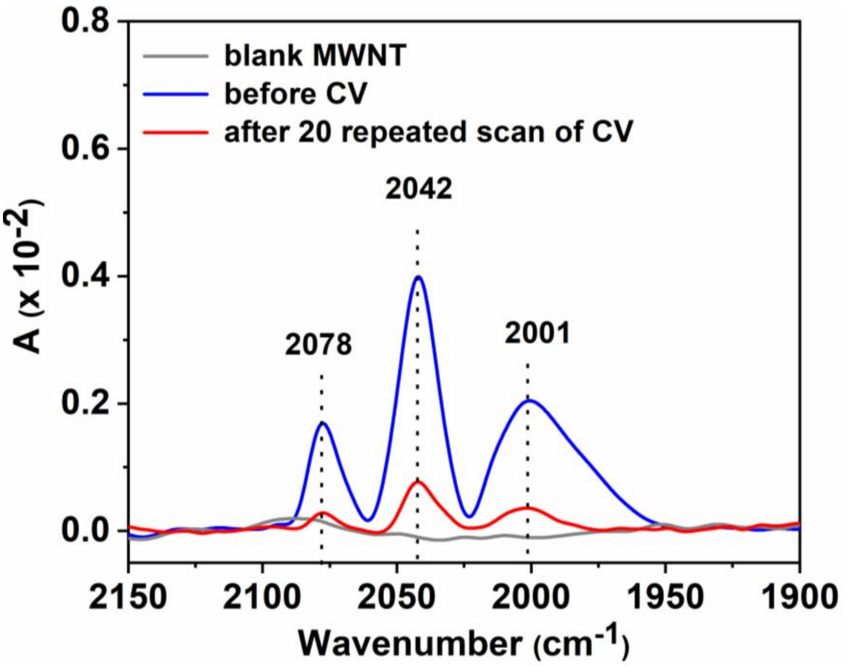

Figure S17: Stability of **2**/MWNT during repeated CV scans using ATR-FTIR spectroscopy. ATR-FTIR Spectra of fresh **2**/MWNT electrode *vs*. that after twenty repeated cycles (red trace) of CV in 0.2 M phosphate buffer pH 7 at room temperature (ν = 100 mV s^{-1}).

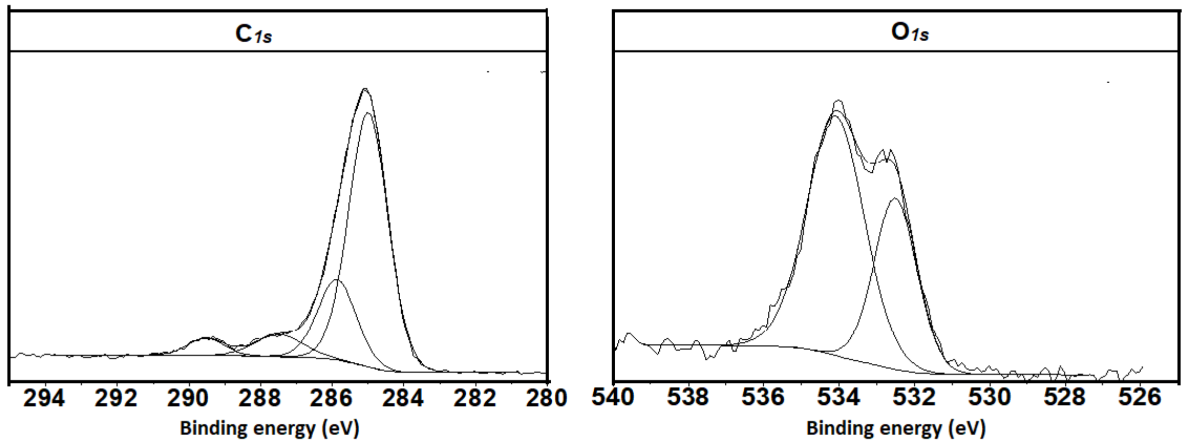

Figure S18: XPS spectra complex **2** showing binding energies of the 1s orbital of C and O.

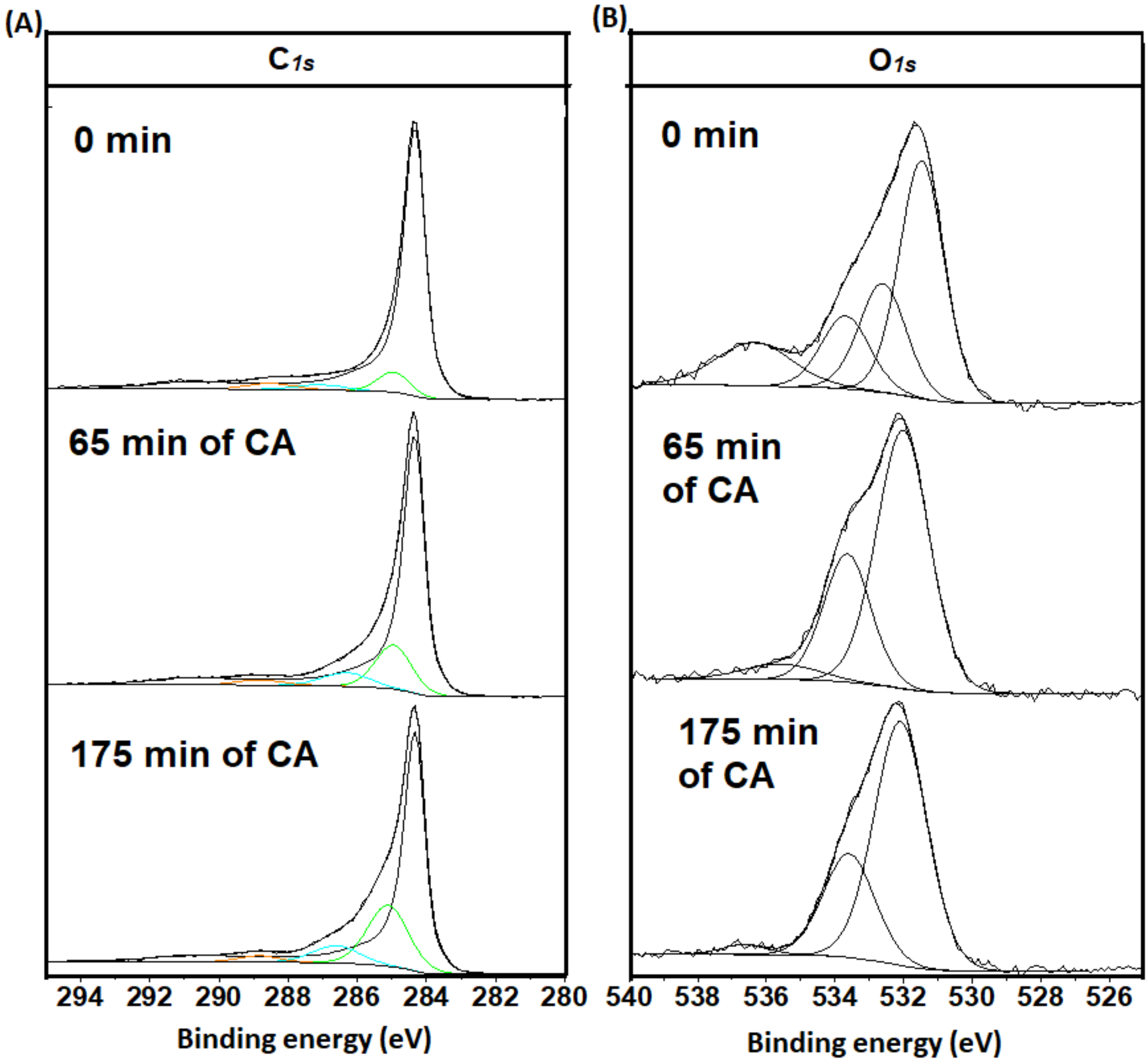

Figure S19: XPS spectra of complex **2**/MWNT. The spectra show: (A) 1s orbital of C; (B) 1s orbital of O of complex **2**/MWNT electrode surface at different interval (0, 65, 175 min) of CA at –0 .49 V *vs.* RHE in 0.2 M sodium phosphate buffer pH 7 under argon at room temperature.

Table S4: Quantitative analysis of XPS study shown in Figure 6D and S19.

	State of the film	C (nanotubes) %	C (catalyst) %	O %	Fe %	S %
**2**/MWNT	Before electrolysis	72.74	13.84	8.69	0.56	0.69
	After 65 min of electrolysis	69.83	21.64	5.2	0.35	0.88
	After 175 min of electrolysis	60.05	28.95	6.47	0.21	0.73

Table S5: List of electrochemical hydrogen evolution reaction (HER) for analogous heterogenous system functional under aqueous condition.

Catalyst	support	electrolyte	Applied potential *vs.* RHE	TON	Ref.
(μ-$S_2(CH_2)_2$NAr) $[Fe(CO)_3]_2$	Pyrolytic graphite electrode	0.5 N H_2SO_4	−480 mV	>10^8	[5]

(μ-$S_2(CH_2)_2$NAr) $[Fe(CO)_3]_2$	Pyrolytic graphite electrode	0.2N H_2SO_4	−658 mV	>10^7	[6]
(μ-$S_2(CH_2)_2$NAr) $[Fe(CO)_3]_2$	Pyrolytic graphite electrode	pH 5.5	−375 mV	0.64 x 10^6	[7]
Ni[P$(CH_2)_2$NAr$]_4$	Carbon nanotube	0.5 M H_2SO_4	−280 mV	10^5	[8]
Co(DO)(DOH) -pnCl_2	Carbon nanotube	pH 4.5	−590 mV	5.5 x 10^4	[9]
Complex **2**	Carbon nanotube	pH 7	−490 mV	~0.6 x 10^4	[This work]

Here, Ar = aromatic functional group, ((DOH)$_2$pn) = N_2, N_2-propanediylbis (2,3butanedione 2-imine 3-oxime.

Table S6: X-ray crystallographic data for complex 2.

**Compound**	**Complex 2**
Formula	C58H38Cl4.04Fe2O10S2
Dcalc./ g cm^{-3}	1.536
m/mm^{-1}	0.899
Formula Weight	1213.92
Color	translucent intenseorange
Shape	needle
Size/mm^3	0.45×0.12×0.07
T/K	150.2(3)
Crystal System	triclinic
Space Group	*P*-1
a/Å	8.4577(3)
b/Å	15.9527(5)
c/Å	20.4022(7)
a/˚	104.959(3)
b/˚	96.529(3)
g/˚	94.589(3)
V/Å^3	2624.99(17)
Z	2
Z'	1
Wavelength/Å	0.71073
Radiation type	Mo Ka
Q*min*/˚	2.087
Q*max*/˚	30.507
Measured Refl.	27936
Independent Refl.	15821
Reflections with I >2(I)	9933
Rint	0.0454
Parameters	863
Restraints	42

Largest Peak	0.791
Deepest Hole	-0.706
GooF	1.020
wR2 (all data)	0.1227
wR2	0.1027
R1 (all data)	0.1106

Table S7: Fractional Atomic Coordinates (×10^4^) and Equivalent Isotropic Displacement Parameters (Å^2^×10^3^) for Complex 2. *Ueq* is defined as 1/3 of the trace of the orthogonalized *Uij*.

Atom	x	y	z	*Ueq*
Fe1	7561.0(4)	1426.4(3)	4824.7(2)	22.4(1)
Fe2	9422.4(4)	1637.3(3)	4010.3(2)	21.96(9)
S1	9304.2(7)	2641.2(4)	5013.4(3)	20.04(14)
S2	6777.9(8)	1814.5(4)	3848.9(3)	21.84(14)
O1	9362.9(19)	4653.8(11)	5568.3(8)	19.4(4)
O2	8284(2)	3999.4(14)	6296.2(10)	31.9(5)
O3	4755.8(19)	3077.1(12)	3312.3(9)	20.7(4)
O4	6622(2)	3150.6(15)	2622.7(10)	37.4(5)
O51	5883(3)	-324.1(16)	4201.6(13)	60.6(7)
O52	9756(3)	706.8(16)	5696.6(12)	47.4(6)
O53	5352(3)	2145.9(14)	5788.4(12)	42.5(6)
O54	12458(3)	1179.0(16)	4589.3(12)	45.8(6)
O55	10767(3)	2703.9(16)	3183.3(12)	46.3(6)
O56	8734(3)	-112.0(15)	3051.4(11)	43.7(6)
C1	8036(3)	3357.4(16)	4737.6(12)	16.9(5)
C2	8174(3)	4235.6(17)	5027.9(12)	17.2(5)
C3	7185(3)	4781.5(17)	4745.2(13)	18.3(5)
C4	7359(3)	5693.6(18)	5018.2(15)	22.3(6)
C5	6445(3)	6198.3(19)	4711.7(15)	26.2(6)
C6	5329(3)	5813.8(19)	4130.5(16)	26.8(6)
C7	5126(3)	4926.7(19)	3858.1(15)	22.5(6)
C8	6055(3)	4385.7(17)	4156.2(13)	18.5(5)
C9	5920(3)	3462.1(17)	3880.4(12)	17.8(5)
C10	6868(3)	2963.0(17)	4164.0(13)	18.6(5)
C11	9344(3)	4458.6(18)	6185.9(13)	21.6(5)
C12	10825(3)	4896(2)	6666.9(14)	24.6(6)
C13	10603(4)	5000(2)	7411.0(14)	28.4(6)
C14	9606(4)	5752(2)	7664.1(15)	29.3(6)
C15	9046(3)	5768.1(19)	8344.6(15)	29.4(6)
C16	7464(4)	5490(2)	8363.2(18)	36.0(7)
C17	6906(4)	5479(2)	8982(2)	42.1(9)

C18	7936(4)	5736(2)	9594.3(18)	40.5(8)
C19	7420(6)	5730(2)	10249(2)	50.5(10)
C20	8455(6)	5966(2)	10830(2)	57.9(12)
C21	10096(5)	6241(2)	10845.1(17)	47.4(10)
C22	11222(7)	6518(3)	11450.2(19)	62.5(13)
C23	12773(7)	6789(3)	11439(2)	65.6(12)
C24	13334(5)	6791(2)	10835.2(18)	51.4(10)
C25	12279(4)	6531(2)	10214.3(16)	38.0(8)
C26	12802(4)	6574(2)	9576.6(16)	34.6(7)
C27	11781(4)	6343.4(19)	8988.9(15)	29.2(6)
C28	10119(3)	6035.1(18)	8959.9(14)	27.1(6)
C29	9573(4)	6008.7(18)	9589.5(15)	30.9(7)
C30	10651(4)	6253.8(19)	10211.0(15)	37.2(8)
C31	5267(3)	2947.7(17)	2682.2(13)	21.1(5)
C32	3923(3)	2544(2)	2117.7(14)	23.9(6)
C33	4405(3)	2475(2)	1413.4(14)	27.0(6)
C34	3026(4)	2116(2)	824.5(15)	28.8(6)
C35	2324(3)	1197.7(18)	769.5(13)	24.5(6)
C36	3131(4)	506(2)	451.0(15)	31.8(7)
C37	2587(4)	-347(2)	383.9(16)	35.5(7)
C38	1180(3)	-568.1(19)	630.2(14)	29.9(6)
C39	561(4)	-1456(2)	559.1(17)	37.8(8)
C40	-781(5)	-1648(2)	795.1(18)	44.4(9)
C41	-1690(4)	-977(2)	1130.2(16)	37.2(7)
C42	-3123(5)	-1169(3)	1374(2)	53.1(10)
C43	-3957(5)	-506(3)	1683(2)	60.8(12)
C44	-3414(4)	354(3)	1768.7(19)	47.9(9)
C45	-1988(3)	583(2)	1538.0(15)	32.5(7)
C46	-1377(4)	1465(2)	1603.9(16)	33.2(7)
C47	-23(3)	1666(2)	1364.9(15)	27.6(6)
C48	904(3)	1000.9(18)	1029.9(13)	22.5(6)
C49	336(3)	121.0(18)	961.3(13)	23.6(6)
C50	-1115(3)	-92.7(19)	1211.5(14)	27.4(6)
C51	6523(4)	357(2)	4443.8(16)	34.9(7)
C52	8887(4)	997.6(19)	5371.2(15)	30.8(7)
C53	6190(3)	1861.7(19)	5407.8(16)	29.8(6)
C54	11283(4)	1358.7(19)	4363.5(15)	29.7(6)
C55	10237(3)	2284(2)	3493.9(15)	29.4(6)
C56	9011(3)	577(2)	3409.0(15)	29.4(6)
Cl5	2153(19)	2425(10)	7644(8)	134(4)
Cl3	744(2)	2459.6(14)	6992.5(14)	128.8(13)
Cl4	3845(3)	3444(3)	7360(2)	97.8(12)
Cl57	3852(12)	3134(11)	7628(8)	122(4)
Cl58	1787(10)	2623(4)	6463(4)	123(3)
C62	2759(7)	2316(5)	6994(4)	122(2)
Cl1	2656.8(15)	382.7(9)	2703.5(6)	81.5(4)

Table S8: Selected bond Lengths in Å for complex 2.

Atom	Atom	Length/Å
Fe1	Fe2	2.4802(5)
Fe1	S1	2.2598(8)
Fe1	S2	2.2776(8)
Fe1	C51	1.791(3)
Fe1	C52	1.789(3)
Fe1	C53	1.805(3)
Fe2	S1	2.2678(7)
Fe2	S2	2.2743(7)

Fe2	C54	1.793(3)
Fe2	C55	1.806(3)
Fe2	C56	1.796(3)
S1	C1	1.779(2)
S2	C10	1.770(3)
O1	C2	1.390(3)
O1	C11	1.375(3)
O2	C11	1.190(3)
O3	C9	1.402(3)
O3	C31	1.373(3)
O4	C31	1.194(3)
O51	C51	1.134(4)
O52	C52	1.140(3)
O53	C53	1.142(3)
O54	C54	1.137(3)
O55	C55	1.134(3)
O56	C56	1.140(4)

Table S9: Selected bond angles in ° for complex 2.

Atom	Atom	Atom	Angle/°
S1	Fe1	Fe2	56.94(2)
S1	Fe1	S2	81.24(3)
S2	Fe1	Fe2	56.92(2)
C51	Fe1	Fe2	104.57(10)
C51	Fe1	S1	161.43(10)
C51	Fe1	S2	89.78(10)
C51	Fe1	C53	99.29(14)
C52	Fe1	Fe2	98.29(9)
C52	Fe1	S1	90.88(10)
C52	Fe1	S2	154.24(9)
C52	Fe1	C51	90.26(14)
C52	Fe1	C53	100.14(13)
C53	Fe1	Fe2	149.62(9)
C53	Fe1	S1	98.75(10)
C53	Fe1	S2	105.27(9)
S1	Fe2	Fe1	56.63(2)
S1	Fe2	S2	81.13(3)
S2	Fe2	Fe1	57.05(2)
C54	Fe2	Fe1	103.79(9)
C54	Fe2	S1	90.29(9)
C54	Fe2	S2	160.64(9)
C54	Fe2	C55	97.44(13)
C54	Fe2	C56	91.01(13)
C55	Fe2	Fe1	150.19(9)
C55	Fe2	S1	103.19(9)
C55	Fe2	S2	101.39(9)
C56	Fe2	Fe1	98.77(9)
C56	Fe2	S1	154.84(10)
C56	Fe2	S2	89.59(9)
C56	Fe2	C55	101.55(13)
Fe1	S1	Fe2	66.43(2)
C1	S1	Fe1	100.85(9)

C1	S1	Fe2	101.50(8)
Fe2	S2	Fe1	66.03(2)
C10	S2	Fe1	101.47(9)
C10	S2	Fe2	100.56(8)
C11	O1	C2	118.78(19)
C31	O3	C9	115.96(18)
C2	C1	S1	124.1(2)
C2	C1	C10	120.3(2)
C10	C1	S1	115.53(19)
O1	C2	C3	116.8(2)
C1	C2	O1	122.0(2)
C10	C9	O3	120.6(2)
C10	C9	C8	121.3(2)
C1	C10	S2	115.85(18)
C9	C10	S2	124.0(2)
C9	C10	C1	120.2(2)
O1	C11	C12	110.0(2)
O2	C11	O1	122.9(2)
O2	C11	C12	127.1(2)

Table S10: Torsion Angles in ° for Complex 2.

Atom	Atom	Atom	Atom	Angle/°
Fe1	S1	C1	C2	145.66(19)
Fe1	S1	C1	C10	-36.68(19)
Fe1	S2	C10	C1	30.73(19)
Fe1	S2	C10	C9	-150.56(19)
Fe2	S1	C1	C2	-146.45(19)
Fe2	S1	C1	C10	31.21(19)
Fe2	S2	C10	C1	-36.70(19)
Fe2	S2	C10	C9	142.01(19)
S1	C1	C2	O1	1.1(3)
S1	C1	C2	C3	175.25(17)
S1	C1	C10	S2	3.8(2)
S1	C1	C10	C9	-174.97(18)
O1	C2	C3	C4	-2.6(3)
O1	C2	C3	C8	174.5(2)
O1	C11	C12	C13	158.3(2)
O2	C11	C12	C13	-22.4(4)
O3	C9	C10	S2	1.5(3)
O3	C9	C10	C1	-179.9(2)
O3	C31	C32	C33	174.3(2)
O4	C31	C32	C33	-5.1(4)
C1	C2	C3	C4	-177.1(2)
C1	C2	C3	C8	0.1(3)
C2	O1	C11	O2	-6.1(4)
C2	O1	C11	C12	173.3(2)

C2	C1	C10	S2	-178.45(18)

References

[1] J. Chen, A. K. Vannucci, C. A. Mebi, N. Okumura, S. C. Borowski, M. Swenson, L. T. Lockett, D. H. Evans, R. S. Glass, D. L. Lichtenberger, *Organometallics* **2010**, *29*, 5330–5340.

[2] G. M. Sheldrick, *Acta Crystallogr A Found Crystallogr* **2008**, *64*, 112–122.

[3] O. V. Dolomanov, L. J. Bourhis, R. J. Gildea, J. A. K. Howard, H. Puschmann, *J Appl Crystallogr* **2009**, *42*, 339–341.

[4] G. M. Sheldrick, *Acta Crystallogr C Struct Chem* **2015**, *71*, 3–8.

[5] S. Dey, A. Rana, S. G. Dey, A. Dey, *ACS Catal.* **2013**, *3*, 429–436.

[6] M. E. Ahmed, S. Dey, B. Mondal, A. Dey, *Chem. Commun.* **2017**, *53*, 8188–8191.

[7] M. E. Ahmed, S. Dey, M. Y. Darensbourg, A. Dey, *J. Am. Chem. Soc.* **2018**, *140*, 12457–12468.

[8] A. Le Goff, V. Artero, B. Jousselme, P. D. Tran, N. Guillet, R. Métayé, A. Fihri, S. Palacin, M. Fontecave, *Science* **2009**, *326*, 1384–1387.

[9] E. S. Andreiadis, P.-A. Jacques, P. D. Tran, A. Leyris, M. Chavarot-Kerlidou, B. Jousselme, M. Matheron, J. Pécaut, S. Palacin, M. Fontecave, V. Artero, *Nat. Chem.* **2013**, *5*, 48–53.
